# Multiple independent acquisitions of a metallophore-synthesis gene by plants through horizontal microbial gene transfer

**DOI:** 10.1038/s41467-025-61162-w

**Published:** 2025-09-22

**Authors:** L. Dirick, Y. Liu, S. Dong, J. Yu, L. Ouerdane, M. Storti, A. Alboresi, C. Curie, B. Goffinet

**Affiliations:** 1 IPSIM, Université Montpellier, CNRS, INRAE, Institut Agro, Montpellier, France; 2 Key Laboratory of Southern Subtropical Plant Diversity, Fairy Lake Botanical Garden, Shenzhen & Chinese Academy of Sciences, Shenzhen, Guangdong China; 3 BGI-Research, BGI-Wuhan, Wuhan, China; 4 Université de Pau et des Pays de l’Adour, e2s UPPA, CNRS, IPREM-UMR5254, Pau, France; 5 Dipartimento di Biologia, Università di Padova, Padova, Italia; 6 Department of Ecology and Evolutionary Biology, University of Connecticut, Storrs, CT USA

**Keywords:** Phylogenetics, Plant evolution, Molecular evolution

## Abstract

The evolution of land plants is marked by major innovations enhancing their vegetative and reproductive fitness. Despite their extensive adaptations to terrestrial habitats, plants rely on ecological interactions with microbes for various physiological processes. Beyond their role as critical partners in the conquest of, and diversification on land, fungi and bacteria also serve as sources of genetic tools. Analyses of the gene space of land plant model organisms suggest that such transfers are unique and ancient. However here, using genomic data spanning the diversity of mosses, we demonstrate that a metallophore-synthesis gene was acquired independently from distinct microbial donors by at least five plant lineages. Furthermore we find that the first NAS gene acquired by mosses was later replaced by another fungal copy, transferred to another major moss lineage. Such a complex history of acquisition of a gene may reflect a more general pattern of highly dynamic gene exchange across the tree of life.

## Abstract (web summary)

Bacteria and fungi provide plants with enhanced access to mineral nutrients and water, but have also served as a source of genes to succeed on land. This study shows multiple independent transfers of a key gene for metal ion transport (NAS) to various plant lineages, highlighting a complex and dynamic history of gene exchange.

## Introduction

The colonization of land by descendants of aquatic or subareal streptophyte algae began no later than 500 mya^1^. Mineral nutrition of early land plants was likely limited by the thin soil layer, and the lack of extensive absorptive organs^2^. Today 90% of land plants across all lineages, except mosses^3^, rely on mycorrhizal fungi to mine the substrate for minerals and water, and extrapolation of such extant dependence back to the origin of land plants suggests that fungi may have been critical to the ability of plants to colonize land^4,5^. The transition of plants to land and their subsequent diversification on land may further have been facilitated by microbial genes acquired via horizontal transfers from bacteria or fungi^6^. While analyses of the gene space of select model taxa highlight the abundance of microbial genes, inferences on their origin rest on the assumption of homology of putative orthologs, leading to a conservative estimate of the frequency of lateral acquisition of these genes during the diversification of land plants. Whether individual genes may, however, be acquired more than once via horizontal transfer and moreover from distinct donors, remains untested.

The ability to move metal ions across the plant body is critical to fundamental physiological processes, and central to this function in flowering plants is the metabolite nicotianamine (NA), which at physiological pH, forms complexes with transition metal ions and enhances their availability^7^. The synthesis of NA by condensation of three molecules of S-Adenosyl-Methionine is catalyzed in one step by nicotiamine synthase (NAS). The *NAS* gene occurs in angiosperms, where the metallophore NA plays a major role in long distance vascular transport by chelation of transition metals (i.e., Fe, Cu, Zn, Ni, Mn)^7,8^, with effects on floral development, fertility and leaf chlorosis^9^. In plants, the metabolite NA is otherwise only known from the moss *Physcomitrium patens*^10^, which is a priori surprising given that it lacks highly modified vascular tissues^11^. The presence of the *NAS* gene in the two major sister lineages of land plants suggests a deep homology of these putative orthologs. Outside of land plants, the *NAS* gene is only known from some fungi and actinobacteria^12,13^, suggesting that land plants acquired a microbial *NAS* gene via horizontal gene transfer (HGT) early in their evolutionary history. However, initial analyses of the NAS proteins of *Arabidopsis thaliana* and *Physcomitrium patens* revealed similarities to distinct fungal homologs, which is inconsistent with a hypothesis of a shared ancestry and raises the question of the timing of these independent acquisitions.

Based on the screening of the NCBI databases and over a hundred recently acquired bryophyte genomes, we demonstrate that the *NAS* gene is indeed lacking in the genome of streptophyte algae sister to land plants as well as in those of lineages emerging from the deepest splits of vascular plants (i.e., lycophytes) and bryophytes (i.e., hornworts and liverworts). Our broad phylogenetic sampling of potential donors and recipients reveals that the history of the *NAS* gene in plants is highly complex: it is characterized by multiple independent transfers from diverse microbes to discrete lineages of plants, a loss and replacement of the *NAS* gene during the evolution of mosses and a lateral transfer within mosses. These findings challenges the current assumption of deep homology of microbial genes in plants and suggest highly dynamic transfers of microbial genes during the diversification of plants on land.

## Results and discussion

### Independent transfers of the *NAS* gene to and within mosses

**Fig. 1:**
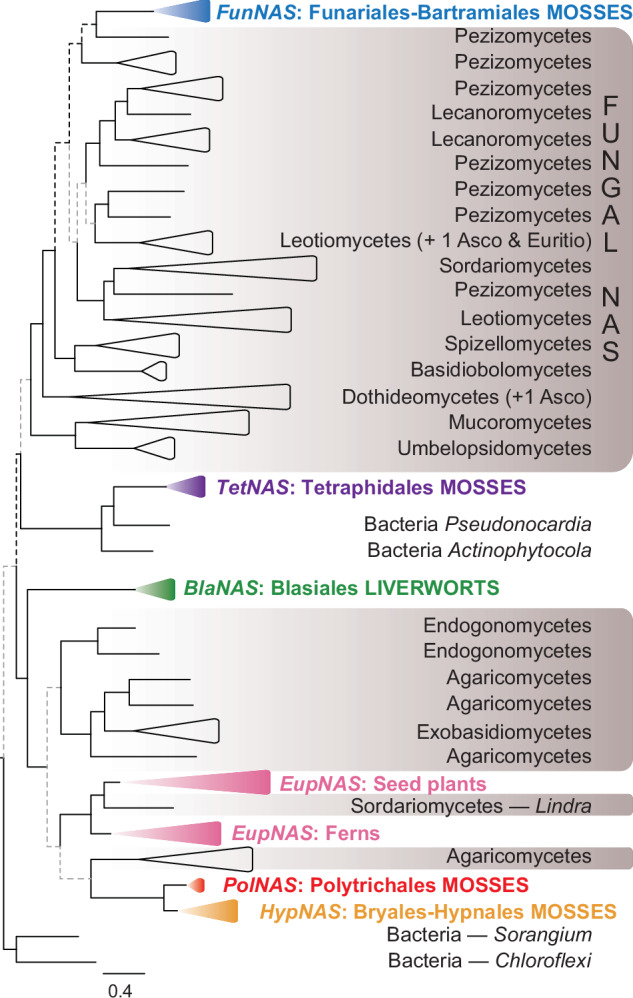
Summary of the evolutionary relationships of NAS genes across the tree of life. *NAS* gene tree, with major clades collapsed, inferred from amino acid sequences of *NAS* genes present in bacteria, fungi, ferns and seed plants (euphyllophytes), liverworts (i.e., only in *Blasia*) and mosses, which hold three distinct copies, the bacterial *TetNAS*, or fungal genes (i.e., *FunNAS* and *HypNAS/PolNAS*). For the full gene tree see Supplementary Fig. 1. *BlaNAS*, *EupNAS*, *FunNAS*, *HypNAS*, *PolNAS* and *TetNAS*: NAS gene typified by the homolog in *Blasia*, Euphyllophytes, *Funaria*, *Hypnum*, *Polytrichum* and *Tetraphis*, respectively.

**Fig. 2:**
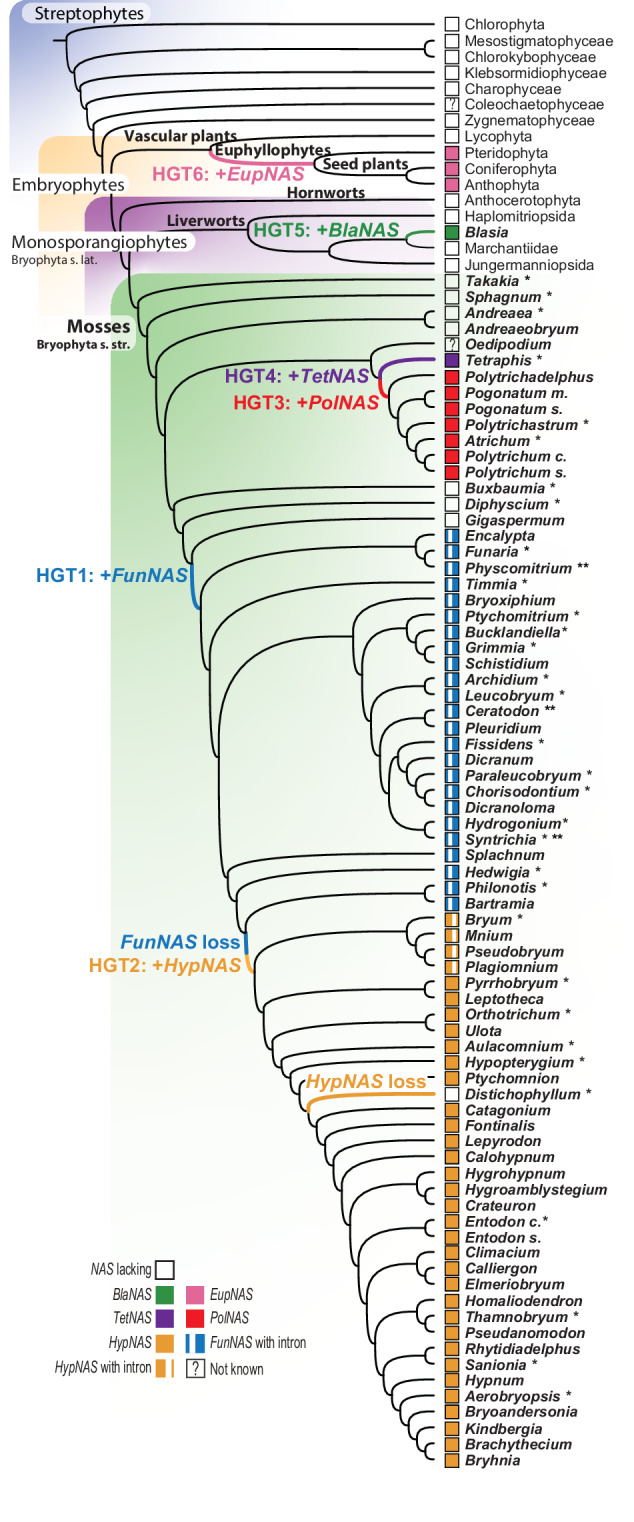
Evolutionary history of the *NAS* gene in land plants. Phylogenetic relationships of land plants drawn from published inferences^16,20^. * following moss or bryophyte names mark accessions for which long sequencing reads (Nanopore) were generated to assess the location of the *NAS* gene in the plant genome. ** refer to data extracted from published genomes (see Methods). All other inferences about the presence or absence of the *NAS* gene in mosses are based on assembled short read data. Filled squares indicate the presence of a *NAS* gene, with the color matching the respective colored branches marking the putative phylogenetic entry of the *NAS* gene during the diversification of mosses. A white vertical bar in the square refers to the presence of an intron in the *NAS* gene, and the location (left in *FunNAS* and right in *HypNAS*) indicating the non-homology of the introns. A question mark (?) identifies the sole supraordinal lineage of mosses (i.e., the monospecific Oedipodiopsida) for which data are not available. *BlaNAS*, *EupNAS*, *FunNAS*, *HypNAS*, *PolNAS* and *TetNAS*: NAS gene typified by the homolog in *Blasia*, Euphyllophytes, *Funaria*, *Hypnum*, *Polytrichum* and *Tetraphis*, respectively.

Broadening the search of the *NAS* gene in mosses beyond the model taxon *Physcomitrium patens*, based on an extensive set of recently generated bryophyte genomes (Supplementary Data 1) reveals that most moss lineages harbor the gene, none of which are, however, a strict homolog of the *NAS* gene present in *Arabidopsis* (Fig. 1; Supplementary Fig. 1). Furthermore, the *NAS* genes in mosses belong to at least three distinct lineages of the *NAS* gene, each more closely related to a different fungal or bacterial donor lineage (Fig. 1; Supplementary Fig. 1), and hence each originating from an independent HGT event. The diversity of *NAS* genes in mosses is robustly structured along the evolutionary history of mosses (Fig. 2). First, the grade of lineages emerging from the earliest splits in the moss phylogeny (i.e., from *Takakia* to *Andreaeaobryum*, Fig. 2) lack the gene altogether, suggesting that it was never part of their gene space. A grade of mosses extending from the Funariales to the Bartramiales harbors a NAS gene (hereafter *FunNAS*; Fig. 2; Supplementary Fig. 2) likely transferred from a Pezizomycete (Supplementary Fig. 1). The gene could not be detected in the Gigaspermales, perhaps due to the partial completeness of its assembled genome (BUSCO 69%^14^). Hence, mosses may have acquired the *FunNAS* prior to the split of the Gigaspermales, but not sooner, since the highly complete genomes of *Buxbaumia* and *Diphyscium* (BUSCO of 97% each^14^) lack the gene (Fig. 2). Those mosses that arose following the divergence of the Bartramiales lack the *FunNAS* but hold, however, a distinct NAS gene (i.e., *HypNAS*; Fig. 2), likely acquired from an Agaromycete (Supplementary Fig. 1). The *HypNAS* is phylogenetically derived from a *NAS* of a distinct fungal donor (Fig. 1) and hence arose from a second HGT replacing the *FunNAS* (Fig. 2). Such phylogenetically correlated loss and gain, i.e., replacement of a specific horizontally transferred gene is undocumented, and highlights, in the case of *NAS*, the strong selection for the acquisition and retention of this gene function during the diversification of mosses. Indeed only a single species, i.e. of the genus *Distichophyllum* representing the derived order Hookeriales, lacks the *NAS* gene. Given the high quality of its genome assembly (i.e., a BUSCO score of 96% based on the Viridiplantae odb10 data set^14^), the absence of the *NAS* gene is best explained by a secondary loss of the *HypNAS* in *Distichophyllum* (Fig. 2). Given that the *FunNAS* is located on the homolog of chromosome 8 of *Physcomitrium patens* and the *HypNAS* on that of a homolog of chromosome 21, the replacement unlikely resulted from a direct homologous recombination. Instead, we hypothesize that the ancestor of the Bryidae, which would have had a *FunNAS*, first acquired the *HypNAS* and then lost the *FunNAS*. Evidence of pseudogenization of the *FunNAS* in any Bryidae genome is lacking, most likely due to the loss dating back to about 200 million years ago^15^.

**Fig. 3:**
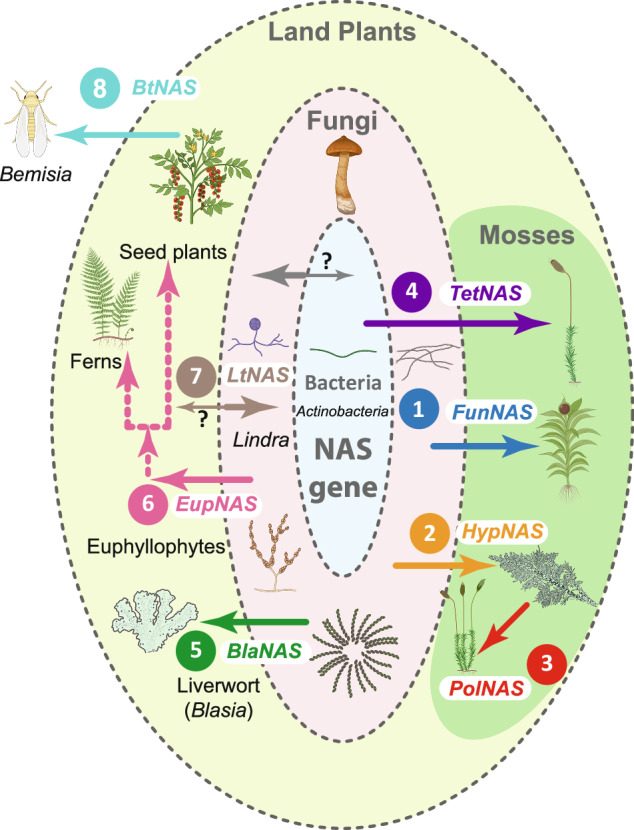
Summary of horizontal gene transfers of the *NAS* gene across the tree of life. Diagram summarizing all eight inferred HGT of the *NAS* gene, based on an evolutionary of the NAS gene rooted with Actinobacteria. The HGT events are numbered only to distinguish them, not to imply a chronological order of their occurrence. Dotted vertical arrows refer to vertical transmissions during the diversification of the lineage, and horizontal arrows refer to putative lateral transfers between bacteria, fungi, plants and insects (i.e., *Bemisia*), and one of the two possible scenarios of the moss to moss HGT (*HypNAS* and *PolNAS*; see Supplementary Fig. 3). Thinner arrows refer to potential alternative transfer polarity. Icons are from BioRender.com and ref. ^65^. *BlaNAS*, *EupNAS*, *FunNAS*, *HypNAS*, *PolNAS* and *TetNAS*: NAS gene typified by homolog in *Blasia*, Euphyllophytes, *Funaria*, *Hypnum*, *Polytrichum* and *Tetraphis*, respectively.

A homolog of the *HypNAS* is also present in the Polytrichales (*PolNAS*, Fig. 2, Supplementary Fig. 1, 3), a more deeply rooted lineage in the moss tree of life (Fig. 2). The *HypNAS* and *PolNAS* genes compose reciprocally monophyletic gene lineages (Fig. 1, Supplementary Fig. 1), suggesting a single origin from a unique donor followed by a moss-to-moss HGT (Fig. 3; Supplementary Fig. 3), i.e., a third *NAS* HGT in mosses. Given this sister-group relationship of the *HypNAS* and *PolNAS*, the polarity of the moss to moss transfer is uncertain but the gene must have been transferred between the ancestor of the sampled Polytrichales and that of the lineage sister to the Bartramiales, immediately after its acquisition and prior to their respective diversification, unless two parallel transfers from a single donor are invoked. An earlier origin of the *PolNAS* in mosses is unlikely given that three lineages that arose after the Polytrichopsida and that compose a grade subtending the remaining mosses (i.e., Buxbaumiales, Diphysciales and Gigaspermales), lack this, and in fact any, NAS gene (Fig. 2). Finally, the Tetraphidales, the sister-group to the Polytrichales^16^, hold a distinct *NAS* gene (*TetNAS*, Fig. 2), which, unlike the *FunNAS* and *HypNAS/PolNAS* is of bacterial rather than fungal origin (Fig. 1, Supplementary Fig. 1). The phylogenetic structuring of the diversity of moss *NAS* genes reveals that the recovery of NAS genes in our data is not due to contamination, and thus reflects at least three independent HGTs from microbes and likely one additional moss-to-moss transfer. Such pattern of independent introductions of microbial genes in these plant genomes, suggests that these genomes are rather permeable to introductions but that the stochasticity of the insertion and/or strong selection prevent microbial genes from accumulating freely.

In all moss samples yielding a NAS metallophore, their genome harbors the fungal or bacterial *NAS* gene flanked by two unambigous plant genes, supporting their effective integration (Supplementary Fig. 4), and hence rejecting the possibility of a fungal contamination in the gene set. The phylogenetic relationships among mosses inferred from variation in their *NAS* genes (Supplementary Fig. 2, 3) reflect current concepts of diversification of these lineages^17^, highlighting the occurrence of the *NAS* gene in the germ line and its vertical transmission, which further supports their effective integration in the genomes^18^. The three distinct *NAS* genes in mosses are also each characterized by distinct predicted motifs that are conserved within their respective lineages, as well as highly congruent with those of the potential donors (Supplementary Fig. 5). Effective integration and functionalization of the *NAS* genes is further substantiated by their expression based on recovery of transcripts from public databases (Supplementary Fig. 6a) and our detection of the metabolite (Supplementary Fig. 7), whose structure is identical to that of the flowering plants (Supplementary Fig. 8).

The retention (and in one case replacement) of the *NAS* gene throughout much of the evolution of mosses suggests that this gene performs physiological functions critical to the fitness of the plant. In *Physcomitrium patens* the gene is expressed primarily in the rhizoids and the protonema, and to a lesser extent in the early stages of sporophyte ontogeny (Supplementary Fig. 6b). Knocking out the single copy *FunNAS* of *Physcomitrium patens* (*PpNAS;* Supplementary Fig. 9) slowed down its growth (Supplementary Fig. 10A, B), decreased maximum efficiency of photosystem II (F_v_/F_m_; Supplementary Fig. 10C), and lack of sexual organogenesis, paralleling the effects of *NAS* inactivation in flowering plants^9,19^.

### Independent transfers of *NAS* to plants

The *NAS* gene is functionally well studied in model angiosperms, including *Arabidopsis*^19^, but its evolutionary history remains unexplored. Its detection in the moss *Physcomitrium patens*^10^ suggests a shared ancestry of the gene, given that vascular plants and monosporangiophytes, or broadly speaking bryophytes, are now considered diverging lines of evolution emerging from a common ancestor^1,20,21^. However, in addition to demonstrating that mosses acquired the gene only after their early diversification (Fig. 2), we could not identify a *NAS* gene in available genomes of hornworts^22,23^, or of the model liverwort *Marchantia polymorpha*^24^, which supports the absence of the *NAS* in ancestral bryophytes. We confirmed these absences by screening available genome assemblies of hornworts and liverworts (Supplementary Data 1). We discovered a *NAS* gene in one liverwort only, namely *Blasia pusilla*. This *BlaNAS* gene is flanked by two plant genes in the genome of *Blasia*, and the copy is phylogenetically linked to a yet another microbial donor, distinct from all other plant *NAS* donors, and thus likely resulting from an independent acquisition of the gene in this lineage of three species all characterized by harboring endophytic cyanobacteria^25^. Within the vascular plants, we uncovered the *NAS* gene in the genome of ferns^26^, but not from the recent genome assemblies of lycophytes^27^, suggesting that the vascular plant *NAS* gene (*EupNAS*) was acquired independently in the ancestor to Euphyllophytes (Fig. 2).

The current distribution of *NAS* gene among land plants reveals parallel acquisitions of the *NAS* gene after the divergence of vascular plants and bryophytes and following the initial speciation events within these lineages, a scenario rejecting the hypothesis of a common origin in the early land plants as recently inferred^28^. Furthermore, we could not identify *NAS* gene in any of the genome of streptophyte algae^29–31^, thereby confirming the absence of the gene in the ancestors to land plants. In fact, given the current time tree of land plant diversification^1^, the *EupNAS* gene was acquired by euphyllophytes between 437 and 400 mya, thus earlier than any of the NAS genes by mosses. The comprehensive time tree for mosses^15^ suggests that the *FunNAS* was transferred about 275 mya, and subsequently lost and replaced by the *HypNAS* about 210 mya (Supplementary Fig. 3). Thus, while the survey of the genome of model species representing modern plant diversity suggested that the *NAS* gene was part of a set of genes acquired during one of two episodes of massive gene transfers to Streptophytes^28^, the occurrence of *NAS* genes in only some lineages of land plants, and their respective phylogenetic affinities to fungal genes, in particular, suggests instead that the gene entered the land plant genome on multiple occasions during their diversification and from multiple donors.

Such a pattern of independent HGT of the *NAS* from distinct source lineages extends to fungi. The NAS gene is present in many fungal genomes spanning much of the phylogenetic breadth of fungi. The absence of the gene in some species or lineages was previously proposed to result from independent losses^13^. Our phylogenetic reconstruction of the NAS gene tree resolves some speciose fungal lineages (e.g., Pezizomycetes and Dothideomycetes) as polyphyletic, suggesting that the distribution of the *NAS* in fungi is shaped, in addition to potential secondary losses, by HGT across the fungal tree (Fig. 1; Supplementary Fig. 1). Whether independent transfers involving multiple prokaryotic donors contributes to explaining the phylogenetic distribution of the *NAS* gene in fungi remains ambiguous given our scant knowledge of *NAS* distribution among bacteria lineages.

The transfer of the *NAS* gene extends further, with plants acting as donor of the gene to a fungus and an insect. We indeed discovered a single fungus, the marine sordariomycete *Lindra*, holding a *NAS* gene that is distantly related to the clade of *NAS* genes of all other sordariomycetes and even of all other fungi sampled. The *LtNAS* gene is phylogenetically nested between two *EupNAS* lineages of euphyllophytes, and sister to the *EupNAS* of seed plants (Fig. 1; Supplementary Fig. 1), suggesting a reverse plant-to-fungus HGT (Fig. 3), and adding to a growing number of such transfers^32,33^. Finally, we located a gene (*BtNAS*) of the *EupNAS* gene in the genome of the whitefly *Bemisia tabaci* (Fig. 3), already known to hold other plant genes^34,35^, and which is at present the only metazoan to have integrated a *NAS* gene, moreover via a transfer from an angiosperm, allied to Cucurbitales (Supplementary Fig. 1).

The higher diversity of microbial genes in bryophyte genomes, even between lineages that diverged within the lats 60 million years^36^, suggested already that these may be more prone to effective insertion of foreign genes than vascular plants^14,28^. Such pattern may reflect their weak developmental links that facilitate foreign transfers^37,38^ and the high totipotency and ability of their cells to develop new germ lines^39^, lowering the barrier to HGT that a strong separation of somatic tissue and germ line would otherwise constitute. Mechanisms of HGT between microbes are rather well studied and understood^40^, whereas those underlying transfers to higher eukaryotes remain more ambiguous^41^. The reconstruction of the evolutionary history of the microbial NAS gene in mosses highlights that, however obscure, such mechanisms may not be the limiting factor to effective HGT, and that, if bryophytes are more permeable to foreign genes entering their cells, strong selection limits their integration and accumulation in their genomes. Direct organismal interactions may a priori seem to facilitate fungal-to-plant transfers^32^, and while mosses engage in a variety of interactions with fungi^42^, including endophytic fungi^43^, they are the sole lineage of land plants universally lacking mycorrhizal associations, although the early diverging *Takakia* harbors mycorrhiza-like glomalean fungi^44^. The NAS gene is currently only known, among bryophytes, from species lacking mycorrhizal associations (i.e., mosses and *Blasia*), and may perhaps at least partially compensate for the absence of fungal symbionts by enhancing the capacity and efficiency of the internal distribution of metal ions.

Fungi and bacteria shaped the ability of plants to colonize and diversify on land, through their ecological interactions^45^ and also as a source of genetic tools^46^. The evolution of land plants is characterized by two major bursts of genomic innovation, with various genes likely acquired by horizontal gene transfer from fungi^47^. The phylogenetic history of the *NAS* gene, one of a large suite of genes hypothesized to have been acquired early in the evolution of land plants, reveals that it has been transferred independently from distinct microbial donors to the ancestor of euphyllophytes (ferns and seed plants), to one liverwort and to three distinct lineages of mosses (Fig. 3). Reconstructing the evolution of genes of microbial origin during the diversification of plants within a comprehensive sampling of land plants revealed the potentially highly dynamic nature of such transfers from a suite of donors, and that transferred genes are more frequently becoming fixed, i.e., entering the germ line and integrated to benefit to plant, than might have been previously assumed or accepted^38,48^, as in the case of the squalene-hopene cyclase, transferred independently from bacteria and cyanobacteria to mosses and liverworts and ferns, respectively^26^. HGTs of microbial genes are thus not only phylogenetically and genomically widespread, i.e., occurring throughout the tree of life and pertaining to a wide array of functional genes, but potentially homoplasious and recurrent within major lineages.

## Methods

### Identification and selection of available microbial and plant *NAS* sequences

Fungal *NAS* sequences were retrieved from JGI/Mycocosm (https://mycocosm.jgi.doe.gov/mycocosm/home). Genomes of exemplars of all fungal phyla and subphyla^49^ were searched individually using Blastp with an e value cutoff at e-30. For plant lineages, we screened available database to identify fern *NAS* (https://fernbase.org/)^26^, *Ginkgo NAS* sequences from^50^ and Angiosperm sequences from GenBank (https://www.ncbi.nlm.nih.gov/genbank/)^51^. Displayed angiosperm *NAS* sequences (50) span the angiosperm phylogenetic tree^52^. All moss *NAS* sequences are from this study, except for *Physcomitrium patens* (https://phytozome-next.jgi.doe.gov/)^53,54^, *Ceratodon purpureus* (https://www.ncbi.nlm.nih.gov/protein)^51,55^, *Calohypnum plumiforme*^56^, *Syntrichia caninervis*^57^, *Entodon seductrix*, and *Hypnum curvifolium*^58^.

### Identification of bryophyte NAS sequences

Genome assemblies of 133 bryophytes, including 121 recently acquired accessions derived from the bryophyte genome project^14^ were screened (Supplementary Data 1). Dong et al.^14^ extracted genomes mostly from wild populations of bryophytes and assembled these based on raw data generated through three high throughput sequencing methods and filtered for microbial contamination. The genomes are of high quality and completeness considering their BUSCO^59^ score exceeding 80% (except for the moss *Gigaspermum repens*, 69%, and the liverwort *Ptilidium pulcherrimum*, 76%), with an overall median and average score of 95% and 94%, respectively^14^. The NAS protein sequence of *Physcomitrium patens* (Pp3c8_970) was used as the query to search against the bryophyte genome sequences using NCBI tblastn with an e-value threshold of 1e-10. The contigs containing the putative *NAS* sequences were identified, and the *NAS* gene structure of each species was predicted with GeneWise (https://www.ebi.ac.uk/Tools/psa/genewise/) using the *NAS* gene sequence of *Physcomitrium patens* as a reference. The *NAS* coding sequences were then extracted, translated into amino acids using the standard genetic code, and used for subsequent analyses.

### Phylogenetic analyses of *NAS* gene and identification of conserved motifs

A total of 330 NAS protein sequences, including 87 NAS sequences from 72 mosses and one liverwort, 55 of 27 seed plants, five of two ferns, four of bacteria and 179 of fungal species were aligned using MAFFT v5^60^ with default settings. The resultant alignment was used for phylogenetic reconstructions using the maximum likelihood (ML) method with IQTREE2 v2.2.0^61^ (with the command: iqtree2 -s alignment.phy -bb 1000 -m MF). ModelFinder^62^ as implemented in IQTREE2 to select the best-fit models. Ultra-fast bootstrap (BS) analyses were implemented for 1000 pseudoreplicates. The motifs in the NAS protein sequences were identified with MEME suite^63^ with the classical mode and the number of motifs set to 10.

### Mass-spectrometry analysis

Crude extracts were prepared from fresh tissue harvested for the following species *Sphagnum* *affine* (Goffinet 14663), *Andreaea rothii* F.Weber & D.Mohr (Goffinet 14656), *Tetraphis pellucida* Hedw. (Goffinet 14664), *Atrichum crispulum* Bescherelle (Goffinet 14665), *Polytrichum commune* Hedw. (Goffinet 14652), *Bartramia pomiformis* Hedw. (Goffinet 14653) *Aulacomniun palustre* (Hedw.) Schwägr. (Goffinet 14654) and *Fontinalis antipyretica* Hedw. (Goffinet 14668). All vouchers deposited in conn herbarium. Fresh tissues were frozen in liquid nitrogen and grinded in a mortar; powder was resuspended at 100 mg/ 100 ul of water and heated at 83 °C, shaken for 20 min, centrifuged twice at 14000 g to remove debris and the supernatant was aliquoted and frozen at −50 °C prior to HPLC and Mass spectrometry analysis. Analytical reagent grade chemicals such as acetonitrile, formic acid and ammonia were purchased from Sigma-Aldrich (Saint-Quentin-Fallavier, France). Ultrapure water (18 MΩ.cm) was obtained from a Milli-Q system (Millipore, Bedford, MA). Microbore HILIC separations were performed using an Agilent 1100 capillary HPLC system (Agilent, Tokyo, Japan) equipped with a 100 μl min^-1^ splitter module. ICP-MS detection was achieved using a model 7500cs instrument (Agilent) fitted with platinum cones, 1 mm i.d. injector torch and a T-connector allowing the introduction of 5% O_2_. The HILIC/ICP MS coupling was done via an Isomist interface (Glass Expansion, Melbourne, Vic, Australia) consisting of a 20 ml Cinnabar cyclonic spray chamber cooled to 2 °C and fitted with a 50 μl min^-1^ Micromist U-series nebulizer. The column used for HILIC separation was a TSK gel amide 80 (250 mm × 1 mm i.d., 5 μm) from Tosoh Biosciences (Stuttgart, Germany). Gradient elution, at a flow rate of 50 μl min^-1^, was carried out using eluent A, acetonitrile, and eluent B, 5 mM ammonium formate (pH 5.5). The gradient program was: 0–5 min 10% B, 5–45 min up to 50% B, 45–50 min 50% B, 50–52 min up to 65% B, 52–55 min 65% B, 55–60 min down to 10% B, 60–65 min 10% B. 9 µL-aliquots of the samples were mixed with 1.5 µL of NiNO_3_ at 300 µM and with 20 µL acetonitrile, and centrifuged. A 7-μl aliquot of the supernatant was injected into the HILIC column each time.

For HILIC/ESI-MS analysis, the HPLC systems were connected to an Orbitrap Fusion Lumos Tribrid Mass Spectrometer (Thermo Fisher Scientific, Bremen, Germany). The coupling was achieved via a heated electrospray ionization source (Thermo Fisher Scientific). The ion source was operated either in the positive ion mode at 3.0 kV. The vaporizer temperature of the source was set to 120 °C and the capillary temperature to 280 °C. In full MS mode, the resolution was set at 240,000. The exact mass of Ni-nicotianamine complex (C_12_H_20_N_3_O_6_Ni^+^, 5 ppm mass tolerance) was screened all along analysis time.

### High-pressure liquid chromatography (HPLC) analysis of nicotianamine

For NA extraction, fresh moss tissues were ground in liquid nitrogen and resuspended in H_2_O at 100 mg/ml, heated at 80 °C for 20 min and then centrifuged twice at (18,000 × *g*) for 10 min. Crude extract supernatants were aliquoted and frozen at −80 °C. Aliquots (50 μl) of supernatant were derivatized with an equal volume of a *O*-phthaladialdehyde (OPA) solution (12.8 mg of OPA, 2.5 ml of methanol, 10.5 ml of 0.2 M borate buffer, pH 9.9, containing 0.1 M KCl and 25 μl of mercaptopropionic acid) and were incubated in the dark for one min.

HPLC separation was carried out on a PrepStar Solvant Delivery Module (Agilent Technologies, Santa Clara, USA) using a binary gradient: solvent A, phosphate-citrate buffer pH 3; solvent B, methanol/tetrahydrofuran 96%/4% (v/v) gradient at a flow rate of 0.7 ml min^−1^ on a C18 Nucleodur column (250 mm × 4.6 mm; Macherey-Nagel, Düren, Germany). Gradient parameters were 0–20 min, 0–100% B, 20–22 min, 100% B, 22–25 min, 100–0% B. Fluorescence of OPA derivatives was measured with a ProStar Fluorescence detector (Agilent Technologies; excitation 350 nm; emission 455 nm), and peak identification was carried out using pure chemically synthesized NA (T-Hasegawa Co., Tokyo, Japan) as external standard.

### Fluorcam analysis

Chlorophyll fluorescence was measured directly on *P. patens* clones grown on solid media, using a FluorCam 7 (Photon System Instruments). The maximal quantum yield of PSII was measured on dark-adapted plants as Fv/Fm = (Fm–Fo)/Fm, where Fm is the maximum fluorescence level obtained with a pulse of intense white light and Fo is the initial fluorescence level in the dark.

### Growth and culture conditions

*Physcomitrium patens* was grown in KNOP medium (KH_2_PO_4_ (Sigma 7778-77-0) 250 mg/l; KCl 250 mg/l; MgSO_4_.7H2O (250 mg/l), Ca(NO_3_)_2_.4H_2_0 (1000 mg/l) (Sigma C2756), adjusted to pH5.8 with 1 N NaOH, supplemented with microelements H_3_BO_3_ (Merck 1.12015) 614 mg/l, CuSO_4_·5H_2_O (Sigma C-6283) 55 mg/l MnCl_2_·4H_2_O (Sigma M-3643) 389 mg/l, CoCl_2_·6H_2_O (Sigma C-3169) 55 mg/l, ZnSO_4_·7H_2_O (Sigma Z-0501) 55 mg/l, KI (Sigma P-4286) 28 mg/l, Na_2_MoO_4_·2H_2_O (Sigma S-6646) 25 mg/l) and Fe-citrate pH4.2 at 20 µM. Mosses were grown at 21 °C, long days conditions (18 h light), with light intensity at 120 µmol/m^2^/sec.

### Molecular cloning and moss transformation

Primers were designed to disrupt *NAS* coding sequence with the insertion of the G418 resistance cassette (*nptII*). Genomic DNA from WT strain was used as a template to amplify selected homologous regions (P1 + P2 and P3 + P4). All the PCR products were cloned into BNRf vector for moss transformation using the restriction enzymes (REs) indicated below. Moss protoplasts were used for PEG-mediated heat-shock transformation as previously described^64^ with *NAS* KO construct linearized with PmlI and PacI. After transformation and two rounds of selection on G418, resistant lines were evaluated for correct homologous recombination using P5 + pro35Srev (Left Border, LB) and ter35Sfor + P6 (Right Border, RB) primers. Primers NAS_F and NAS_R were used on cDNA libraries to verify *NAS* expression by RT-PCR. ACTIN2_F and ACTIN2_R were used to control the quality of genomic DNA and cDNA templates. P1, atCACGTGCGCATGTGGGTGGAGAAATT (PmlI); P2, atCTCGAGGCGGCATCATAGTCAACGTT (XhoI); P3, atACTAGTGCTTGTTGTATCCGGTGGTG (SpeI); P4, atTTAATTAACCAATCTTCAGCACGTATCCC (PacI); P5, TTCTTACCTGCCTCGCTGTT; P6, CCACCACCTGTCCTACCTG; pro35Srev, GTGTCGTGCTCCACCATGT; ter35Sfor, CGCTGAAATCACCAGTCTCTCT; NAS_F, CAACGCTGTCGGATGATGCT; NAS_R, ACGCGCACAGGAATTTTTGC; ACTIN2_F, GCGAAGAGCGAGTATGACGAG and ACTIN2_R, AGCCACGAATCTAACTTGTGATG.

### Reporting summary

Further information on research design is available in the Nature Portfolio Reporting Summary linked to this article.

## Supplementary information

Supplementary information

Transparent Peer Review file

Reporting Summary

Description of Additional Supplementary Files

Supplementary Data 1

## Data Availability

All analyzed genomes are publicly available through NCBI and all NAS sequences identified in bryophytes genomes deposited on GenBank; all accession numbers are provided in Supplementary Data 1.
