# Supplementary material for: Multiple independent acquisitions of a metallophore-synthesis gene by plants through horizontal microbial gene transfer
Source: Nat Commun. 2025 Sep 22;16:8339. doi: 10.1038/s41467-025-61162-w (PMC12454661; doi:10.1038/s41467-025-61162-w)
Supplement: Supplementary file 1 — Supplementary information [file 41467_2025_61162_MOESM1_ESM.pdf]

Supplementary files

Multiple independent acquisitions of a metallophore-synthesis gene by plants through horizontal microbial gene transfer.

L. Dirick[1, @*], Y. Liu[2, 3, @], S. Dong[2], J. Yu[3], L. Ouerdane[4], Storti M.[5], A. Alboresi[5], C. Curie[1] & B. Goffinet[6*]

[1]IPSIM, Université Montpellier, CNRS, INRAE, Institut Agro, Montpellier, F-34060 France. [2]Key Laboratory of Southern Subtropical Plant Diversity, Fairy Lake Botanical Garden, Shenzhen & Chinese Academy of Sciences, Shenzhen, Guangdong, China. [3]BGI-Research, BGI-Wuhan, Wuhan 430074, China. [4]Université de Pau et des Pays de l'Adour, e2s UPPA, CNRS, IPREM-UMR5254, Hélioparc, 2, Av. Pr. Angot, 64053 Pau, France. [5]Dipartimento di Biologia, Università di Padova, Via Ugo Bassi 58b 35131, Padova, Italia. [6]Department of Ecology and Evolutionary Biology, 75 NorthEagleville road, University of Connecticut, Storrs CT, 06269-3043, USA. E-mail: leon.dirick@cnrs.fr, bernard.goffinet@uconn.edu

Table of content

Supplementary Fig. 1 | Phylogenetic relationships of bacterial, fungal and land plant *NAS* genes.

Supplementary Fig. 2 | Phylogenetic relationships of mosses harboring the *FunNAS* gene.

Supplementary Fig. 3 | Phylogenetic relationships of mosses harboring the *HypNAS* or *PolNAS* gene.

Supplementary Fig. 4 | Flanking genes of the *NAS* gene in exemplar plants, the fungus *Lindra* and the whitefly *Bemisia*.

Supplementary Fig. 5 | Predicted motives of the NAS gene of bacteria, fungi and plants.

Supplementary Fig. 6 | Occurrence of NAS transcripts in representative mosses, and the pattern of expression in the life cycle of *Physcomitrium patens*.

Supplementary Fig. 7 | HPLC analysis of nicotianamine in moss extracts.

Supplementary Fig. 8 | Mosses with NAS genes originating from distinct HGT donors all contain the *bona fide* nicotianamine metabolite.

Supplementary Fig. 9 | Construction of a *nas* knock-out mutant strain in the moss *Physcomitrium patens*.

Supplementary Fig. 10 | Reduced fitness in *Physcomitrium patens* deleted for the NAS gene.

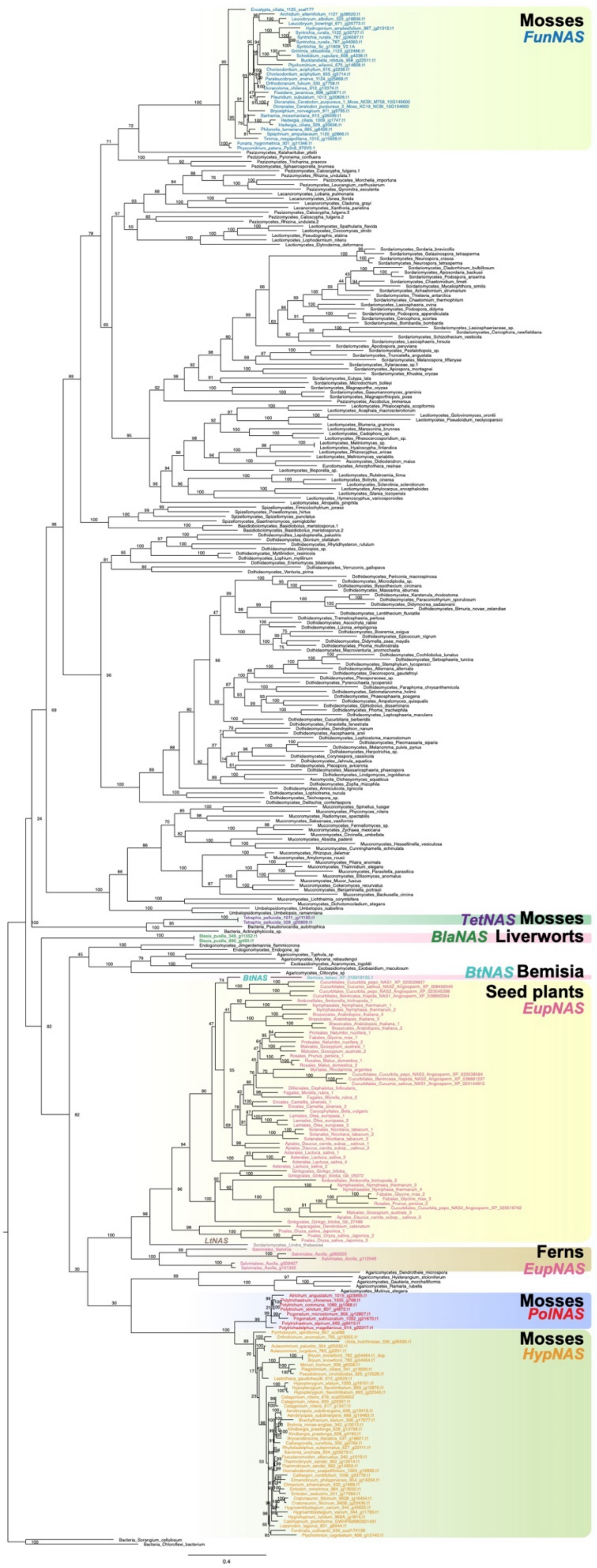

Supplementary Fig. 1 | Phylogenetic relationships of bacterial, fungal and land plant *NAS* gene. Gene tree inferred for the *NAS* gene based on amino acid sequences analyzed under the criterion of maximum likelihood analyses of amino acid sequences using a LG+F+R8 model of evolution. Sequences were sampled from available public database for bacteria known to hold the *NAS* gene, for exemplars of fungal and euphyllophyte lineages and complemented by recently obtained sequences for mosses and one liverwort. Values above branches are bootstrap percentages. *BetNas*, *BlaNAS*, *EupNAS*, *FunNAS*, *HypNAS*, *PolNAS* and *TetNAS*: NAS gene typified by homologue in *Bemesia*, *Blasia*, Euphyllophytes, *Funaria*, *Hypnum*, *Polytrichum* and *Tetraphis*, respectively.

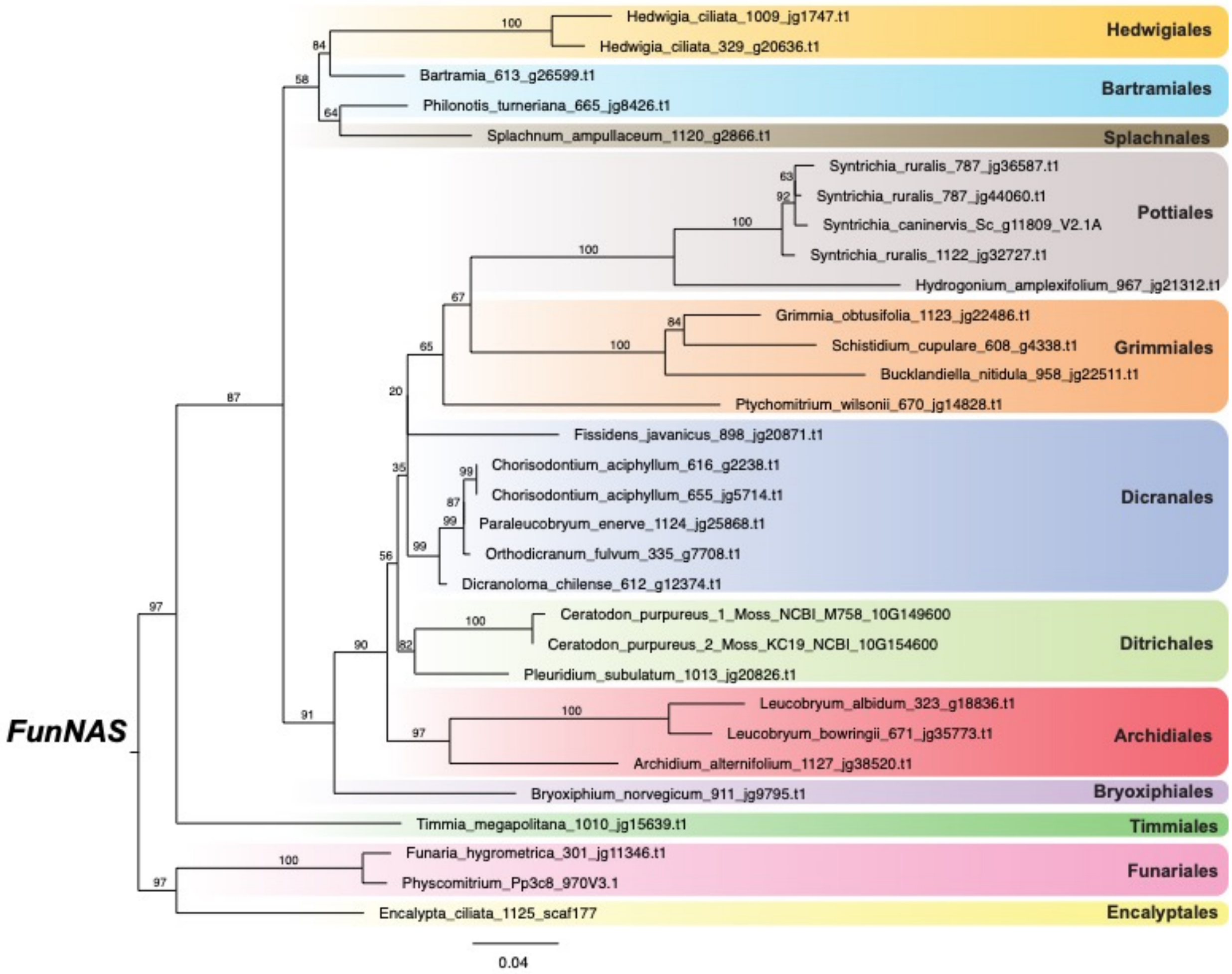

Supplementary Fig. 2 | Phylogenetic relationships of mosses harboring the *FunNAS* gene. Gene tree of *FunNAS* gene inferred from their amino acid sequences, and reflecting the relationships among lineages of Funariidae, Dicranidae and "basal" Bryidae. Values above branches correspond to bootstrap percentages. The *NAS* gene occurs typically as a single copy nuclear gene. The presence in *Hedwigia ciliata* was confirmed extracting genomic DNA twice from a wild population. *Syntrichia*, *Chorisodontium* and *Ceratodon* harbor two or more paralogs, resulting either from tandem duplication, as frequently observed following HGT [1] or from whole genome duplications.

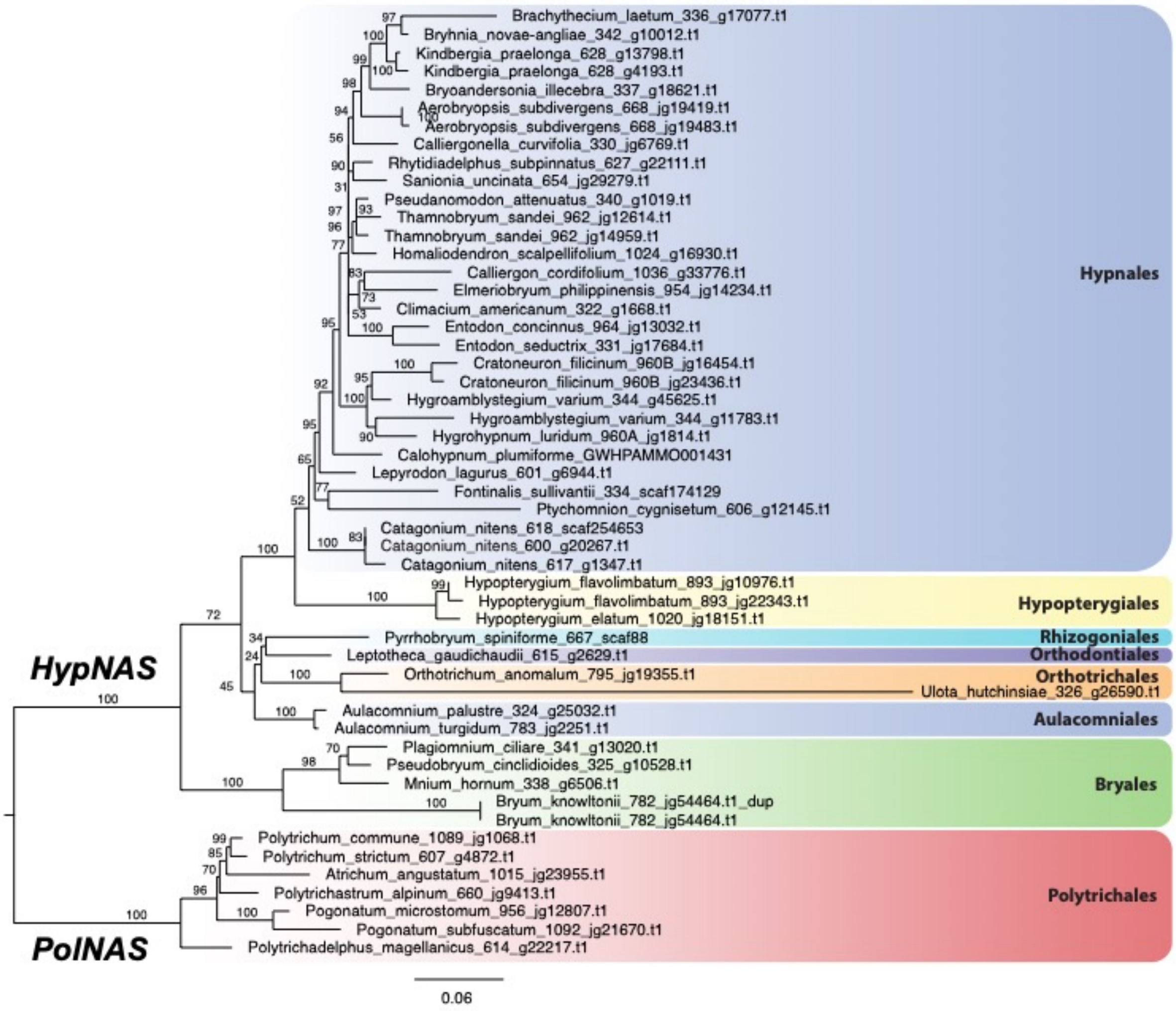

Supplementary Fig. 3 | Phylogenetic relationships of mosses harboring the *HypNAS* or *PolNAS* gene. Gene tree of the *HypNAS* and *PolNAS* gene inferred from their amino acid sequences, and reflecting the sister-group relationship of the clades of lineages harboring the *HypNAS* or the *PolNAS*. This pattern suggests that the *NAS* gene acquired from a funal lineage (Supplementary Figure 1) was transferred from the ancestor of the Bryales and its sister group to that of sampled Polytrichaceae or vice versa. The most recent common ancestor to the extant Polytrichaceae dates back to about 130 mya [2] with the split of the Polytrichopsida from its sister group, the Bryopsida sensu[3] dating back to about 330 mya [2]. The age of the ancestor to the Bryidae bearing a *HypNAS* is about 210 my [2]. Hence, if the *NAS* gene was acquired by the Bryidae and then transferred to the Polytrichales, the origin of the *HypNAS* would date to about 210 mya, and its transfer and hence the origin of the *PolNAS* to about 130 mya. If the *NAS* gene was first acquired by the Polytrichales and then transferred to the Bryidae, the acquisition of the *PolNAS* could date back to the stem age of the lineage (i.e., 330 mya) and no later than 210 mya, when it would have been tranferred to the Bryidae. Both plausible scenarios involve a moss-to-moss HGT, between Bryidae and Polytrichales In all sampled Polytrichaceae, the *PolNAS* is present at a single locus. The number of copies of the *HypNAS* is typically one. *Bryum*, *Aerobryopsis*, *Cratoneuron*,

Kindbergia, *Hygroamblystegium*, *Hypopterygium*, and *Thamnobryum* harbor two or more paralogs, resulting either from tandem duplication, as frequently observed following HGT [1] or from whole genome duplications. In *Hygroamblystegium varium*, for example, two paralogs were recovered, which given their relationship to orthlogs of closely related species, may reflect a whole genome duplication in the ancestor to the Amblystegiaceae, followed by the loss of one paralog in *Hygrohypnum luridum* and *Cratoneuron filicinum.* The latter has two copies likely resulting from a more recent duplication. In *Hypopterygium*, *H. elatum* has one copy, whereas *H. japonicum* has two copies suggesting a loss in the former. Values above branch correspond to bootstrap percentages.

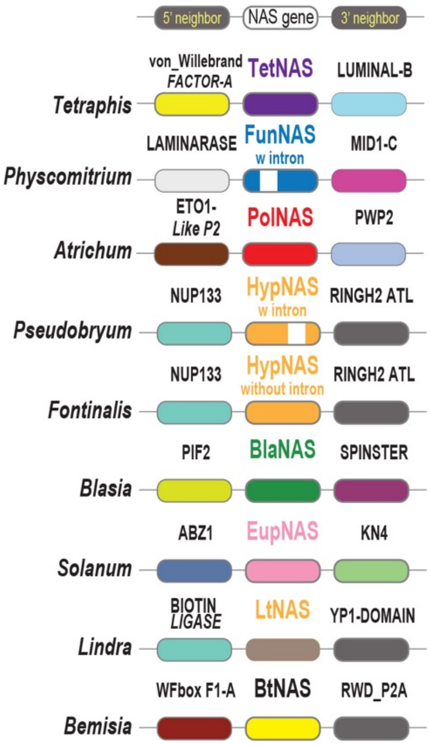

5' neighbor	best homolog	3' neighbor	best homolog
von Willebrand Factor A	*P. patens* LOC112294656	LUMINAL-B	*P. patens* LOC112288048
Laminarase	*P. patens* LOC112286152	MID1-C	*P. patens* LOC112285500
ETO 1	*P. patens* LOC112288611	PWP2	*P. patens* LOC112285031
NUP133	*P. patens* LOC112274272	RINGH2 ATL	*P. patens* LOC112295554
NUP133	*P. patens* LOC112274272	RINGH2 ATL	*P. patens* LOC112295554
PIF2	*Camellia sinensis* LOC114295473	SPINSTER	*Marchantia polymorpha*
ABZ1	*Solanum lycopersi-cum* ID: 544180	KN4	*Solanum lycopersi-cum* ID: 543695
BIOTIN PROTEIN LIGASE	*Trichoderma virens* (Sordariomycetes) GB: EHK23600.1	YP1-DOMAIN PROTEIN	*Colletotrichum graminicola* (Sordariomycetes) GB: EFQ34632.1
WFbox F1-A	*Bemisia tabaci* LOC109044733	RWD domain-containing protein 2A	*Bemisia tabaci* LOC109044679

Supplementary Fig. 4 | Flanking genes of the *NAS* gene in exemplar plants, the fungus *Lindra* and the whitefly *Bemisia*. The nearest functionally annotated 5' and 3' neighoring genes of the various *NAS* genes found in plants (mosses's *TetNAS, PolNAS, FunNAS and HypNAS*, the liverwort Blasia's *BlaNAS*, Euphyllophyte Solanum *EupNAS*), in fungi *Lindra LtNAS* and insect Bemisia *BtNAS*). DNA sequences flanking the corresponding *NAS* gene were translated (http://insilico.ehu.es/translate/) and protein sequences searched using Blastp for the best homolog in public database (https://blast.ncbi.nlm.nih.gov/Blast.cgi); e value< e-30). In all instances, the *NAS* gene was flanked by genes of the host clade, as expected for a *bona fide* HGT integration, and ruling out a potential microbial contamination. The identity of the flanking genes is conserved for each type of moss NAS supporting the hypothesis of a shared ancestral origin of the *FunNAS*, *HypNAS*, or *PolNAS* (see Supplementary Figures 1–3). *BlaNAS*, *EupNAS*, *FunNAS*, *HypNAS*, *PolNAS* and *TetNAS*: NAS gene typified by homologue in *Blasia*, Euphyllophytes, *Funaria*, *Hypnum*, *Polytrichum* and *Tetraphis*, respectively.

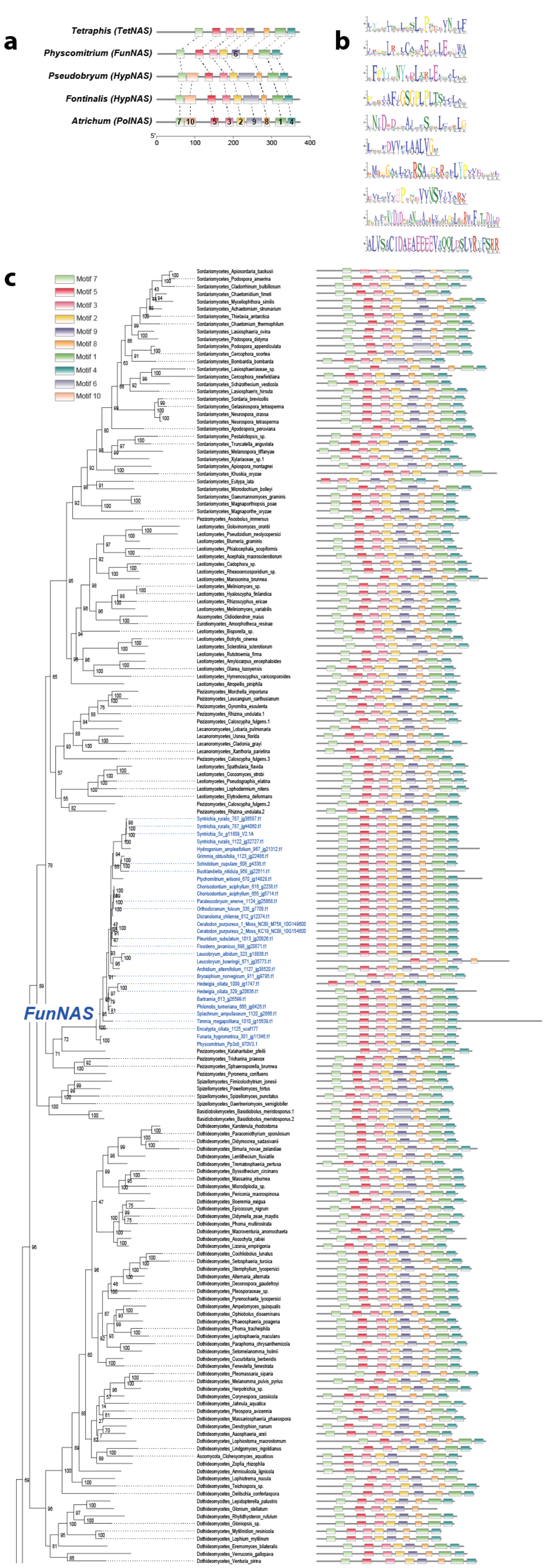

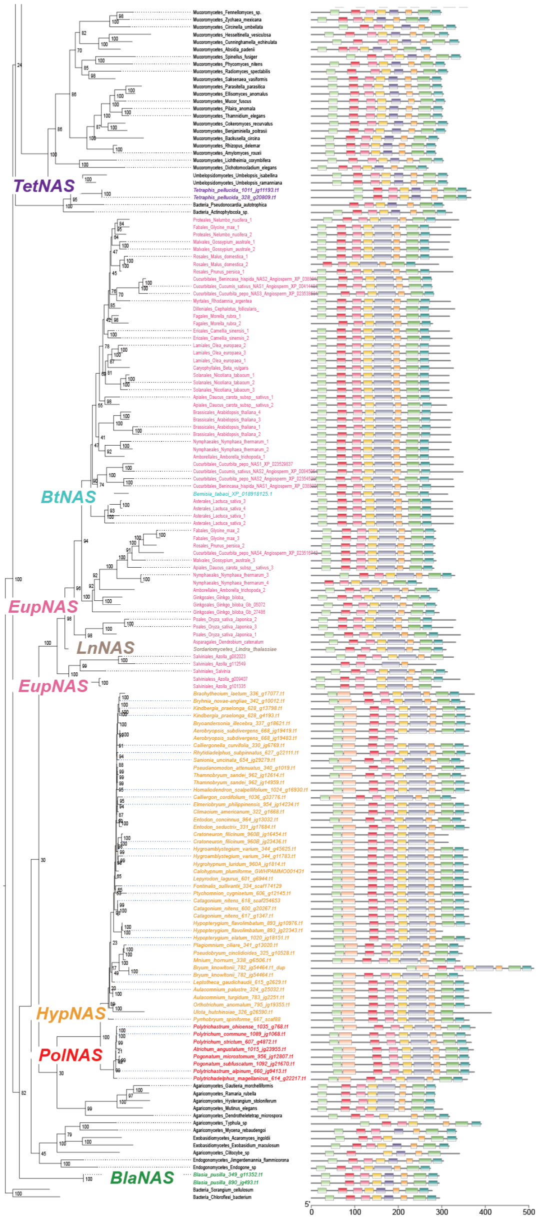

Supplementary Fig. 5 | Predicted motives of the NAS gene of bacteria, fungi and plants.

a, Organization of the motifs of the NAS gene. **b**, Motifs identified by MEME suite, and the probability of amino acid (letter code) at each position in the peptide sequence. **c**) Motifs of all NAS genes sampled for the phylogenetic reconstruction shown in Supplementary Fig. 1. *BetNas*, *BlaNAS*, *EupNAS*, *FunNAS*, *HypNAS*, *PolNAS* and *TetNAS*: NAS gene typified by homolog in *Bemesia*, *Blasia*, Euphyllophytes, *Funaria*, *Hypnum*, *Polytrichum* and *Tetraphis*.

a

Subject Seq-id	Subject Scientific Name	Subject Taxonomy ID	Percentage of identical matches	Alignment length	Number of mismatches	Number of gap openings	Start of alignment in query	End of alignment in query	Start of alignment in subject	End of alignment in subject	Total number of gap	Expect value	Bit score
**gnl|onekp|KEFD_scaffold_2058843**	*Encalypta streptocarpa*	81092	75.472	212	49	3	1	211	1	210	3	4.10E-106	311
**gnl|onekp|ZQRI_scaffold_2018591**	*Timmia austriaca*	108613	75.41	305	66	3	1	304	1	297	9	6.41E-160	451
**gnl|onekp|ABCD_scaffold_2076077**	*Niphotrichum elongatum*	71396	89.457	313	31	1	6	316	1	313	2	0	555
**gnl|onekp|YWNF_scaffold_2006149**	*Hedwigia ciliata*	52988	81.89	127	22	1	89	214	1	127	1	1.83E-67	210
**gnl|onekp|JMXW_scaffold_2040665**	*Bryum argenteum*	37413	37.288	177	104	5	139	313	1	172	7	1.94E-21	92.4
**gnl|onekp|CMEQ_scaffold_2016535**	*Pulvigera lyellii*	61563	35.211	213	129	5	106	313	8	216	9	3.33E-26	106
**gnl|onekp|DHWX_scaffold_2075202**	*Fontinalis antipyretica*	67435	35.814	215	125	5	106	313	8	216	13	8.75E-28	110
**gnl|onekp|QKQO_scaffold_2011320**	*Pseudotaxiphyllum*	186706	34.884	215	127	5	106	313	8	216	13	1.23E-25	105
**gnl|onekp|TAVP_scaffold_2004882**	*Calliergon cordifolium*	671105	34.906	212	127	5	106	313	8	212	11	1.79E-25	104
**gnl|onekp|TMAJ_scaffold_2176874**	*Neckera douglasii*	140378	33.962	159	94	4	106	257	8	162	11	1.75E-16	79

b

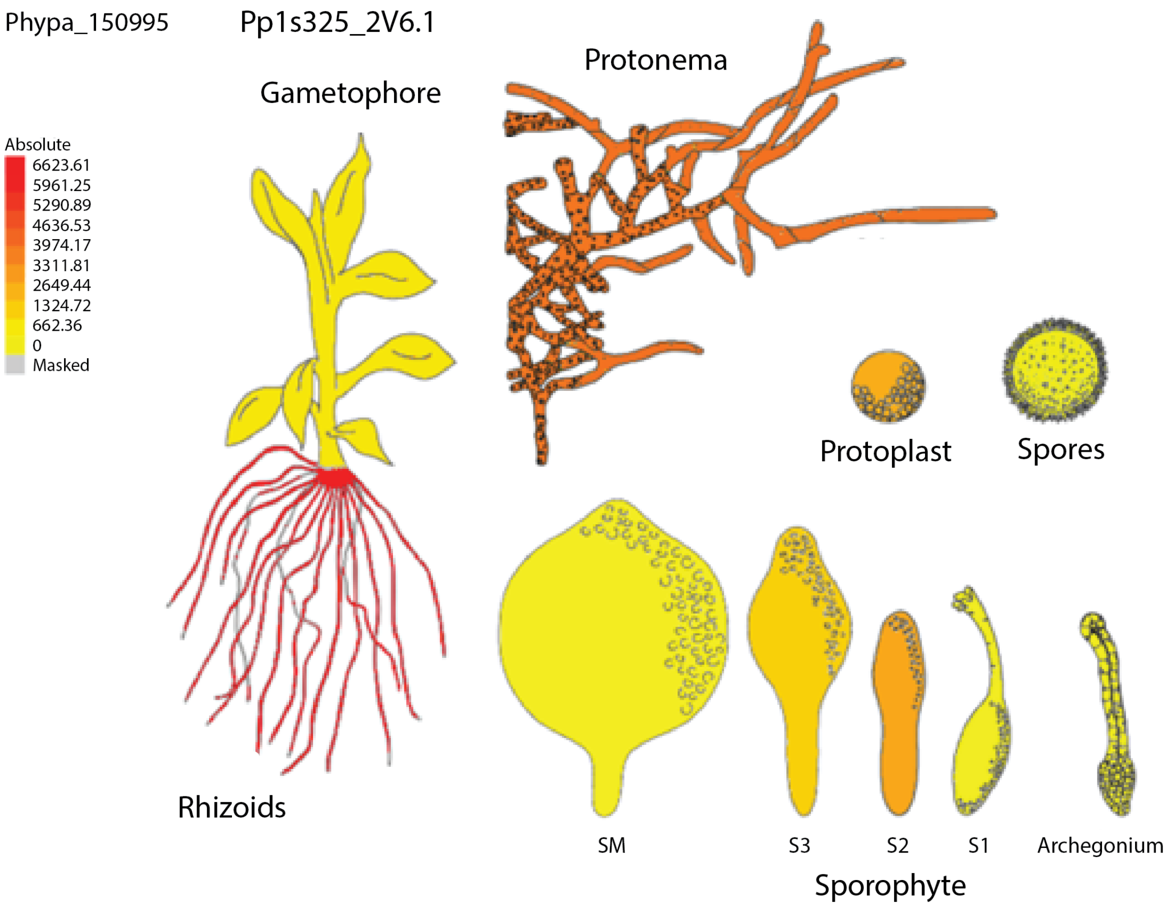

Supplementary Fig. 6 | Occurrence of NAS transcripts in representative mosses, and the pattern of expression in the life cycle of *Physcomitrium patens*. a, Screening of public transcriptomes generated for Bryophyte samples (1KP project, https://db.cngb.org/onekp/) [4], using BLASTP (PpNAS bait) on Bryophyta (taxid 3208) , revealing the presence of a NAS representing the *FunNAS* (*Encalypta*, *Timmia*, *Nipotrichum* and *Hedwigia*) and *HypNAS* (*Bryum*, *Pulvigera*, *Fontinalis*, *Calliergon*, *Pseudotaxiphyllum* and *Neckera*). **b**, Pattern of expression of the *PpNAS* gene (Pp1S325_2V6.1), the ortholog of the *FunNAS* group, in the plant model system *P. patens* (BAR Toronto, http://bar.utoronto.ca/efp_physcomitrella/cgi-bin/efpWeb.cgi) with maximum expression in rhizoids and protonema cells.

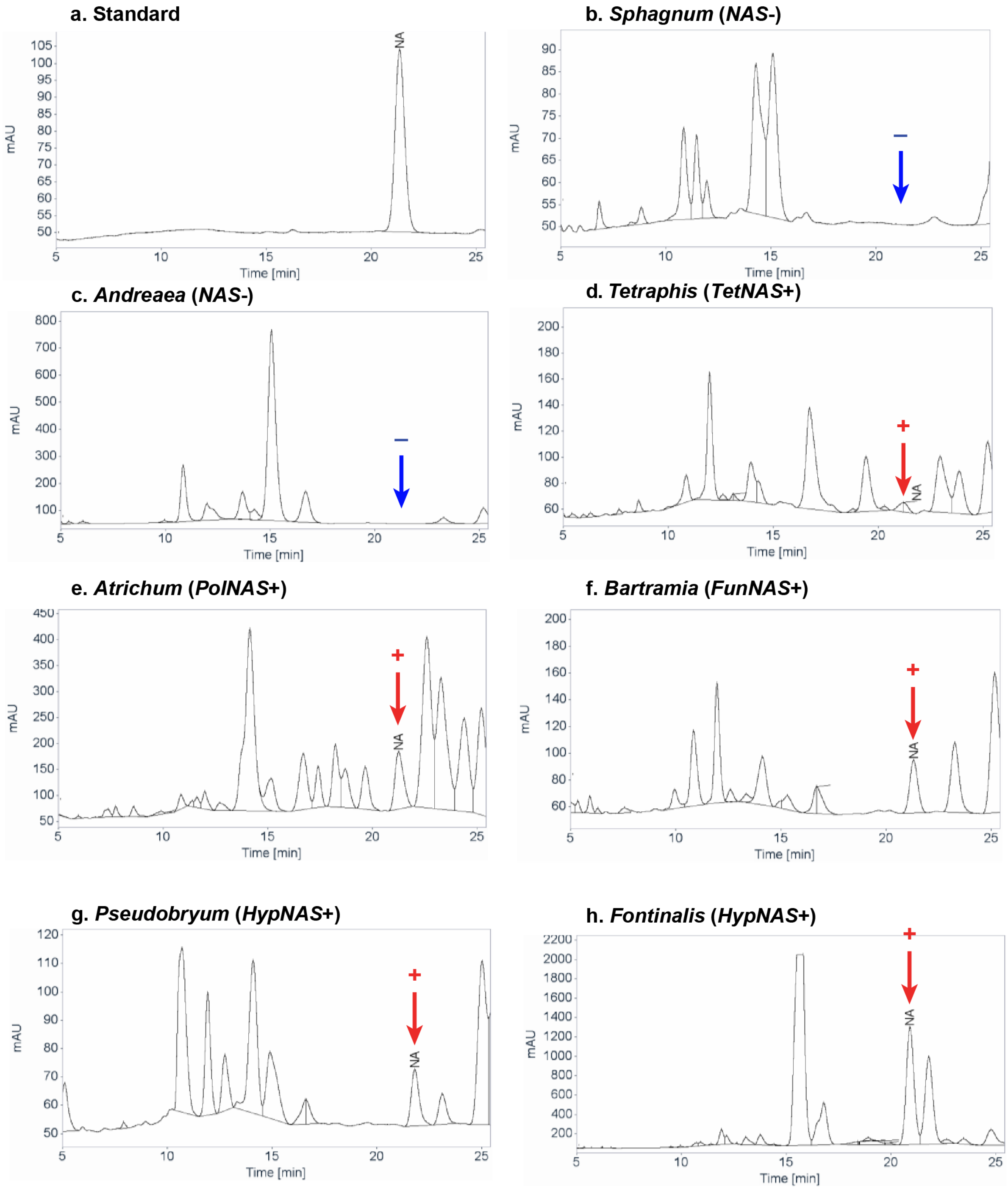

Supplementary Fig. 7 | HPLC analysis of nicotianamine in moss extracts. Survey of exemplars of major orders of mosses for the presence of the metabolite nicotianamine encoded for by the *NAS* gene. **a**, Pure nicotianamine standard serving as reference. **B, c,** *Sphagnum* (**b**)and *Andreaea* (**c**)representing two lineages emerging from deep splits in the moss phylogeny (Fig. 2) and lacking a NAS gene. **d–h**, Exemplars of moss lineages harboring either the *TetNAS* (*Tetraphis*); **d**, *PolNAS* (*Atrichum*); **e**, *FunNAS* (*Bartramia*); **f**, or *HypnNAS* (*Pseudobryum*; **g,** and *Fontinalis*); **h**, gene.

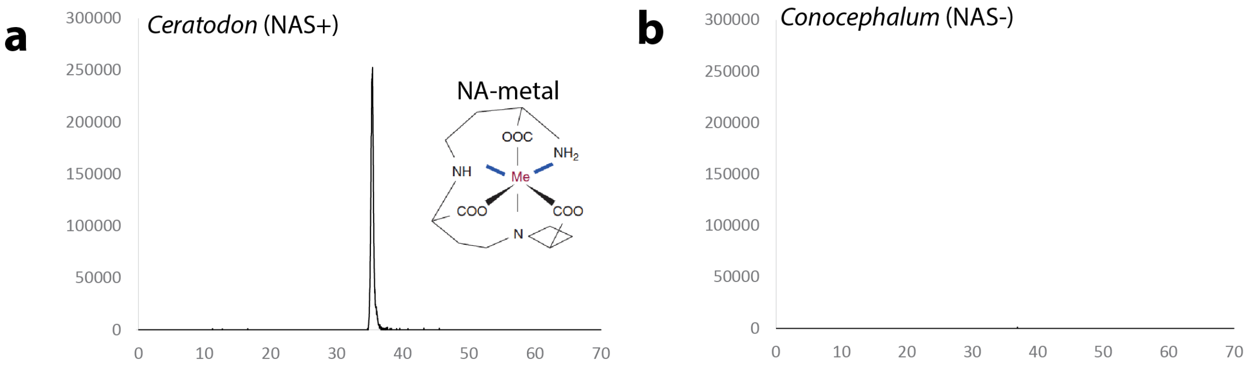

c

Plant sample	Classification	Nicotianamine	NAS orthogroup	Donor
Andreaea	Moss/Andreaeales	-		
Sphagnum	Moss/Sphagnales	-		
Tetraphis	Moss/Tetraphidales	+	TetNAS	Bacteria
Atrichum	Moss/Polytrichales	+	PolNAS	Moss
Ceratodon	Moss/Funariales	+	FunNAS	Fungi
Mnium	Moss/Bryales	+	HypNAS	Fungi
Fontinalis	Moss/Hypnales	+	HypNAS	Fungi
Climacium	Moss/Hypnales	+	HypNAS	Fungi
Conocephalum	Liverwort/Marchantiales	-		
Selaginella	Lycophyte/Selaginalles	-		
Dendrolycopodium	Lycophyte/Lycopodiales	-		

Supplementary Fig. 8 | Mosses with NAS genes originating from distinct HGT donors all contain the *bona fide* nicotianamine metabolite. Detection of nicotianamine by hydrophilic interaction liquid chromatography electrospray tandem mass spectrometry (HILIC ESI MS) coupling. **a,** MS chromatogram showing the exact mass of Nicotianamine-Nickel complex (insert) at 35 min in the moss *Ceratodon purpureus* (*FunNAS*) **b,** MS chromatogram showing reflecting the absence of the metabolite in the NAS- liverwort *Conocephalum conicum*. **c,** Summary of samples analyzed for the presence or absence of the metabolite nicotianamine. All nicotianamine positive mosses samples have identical nicotianamine structures and are identical to nicotianamine in higher plants [5]. Note that the Lycophytes, the sister-group to euphyllophytes contain no nicotianamine, in agreement with the absence of a *NAS* gene in the *Selaginella* genome. *FunNAS*, *HypNAS*, *PolNAS* and *TetNAS*: NAS gene typified by homolog in *Funaria*, *Hypnum*, *Polytrichum* and *Tetraphis*, respectively.

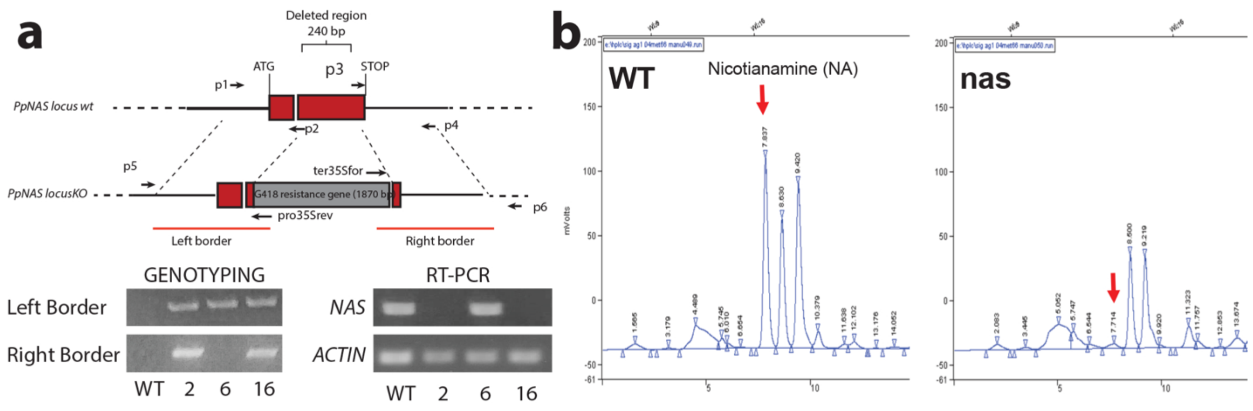

Supplementary Fig. 9 | Construction of a *nas* knock-out mutant strain in the moss *Physcomitrium patens*. **a,** Schematic representation of the deletion in the *FunNAS PpNAS* gene (NCBI PHYPA_010979) by homologous replacement of exon2 with a G418 resistance marker. The deletion marker is correctly inserted in transformed clones 2 and 16 ("Genotyping") and the lack of *NAS* transcripts was verified in the same clones by RT-PCR. Actin is the internal control. Primers are as described in Methods. **b,** HPLC analysis of NA in *P. patens* wildtype (left) and *nasΔ* mutant (right) extracts.

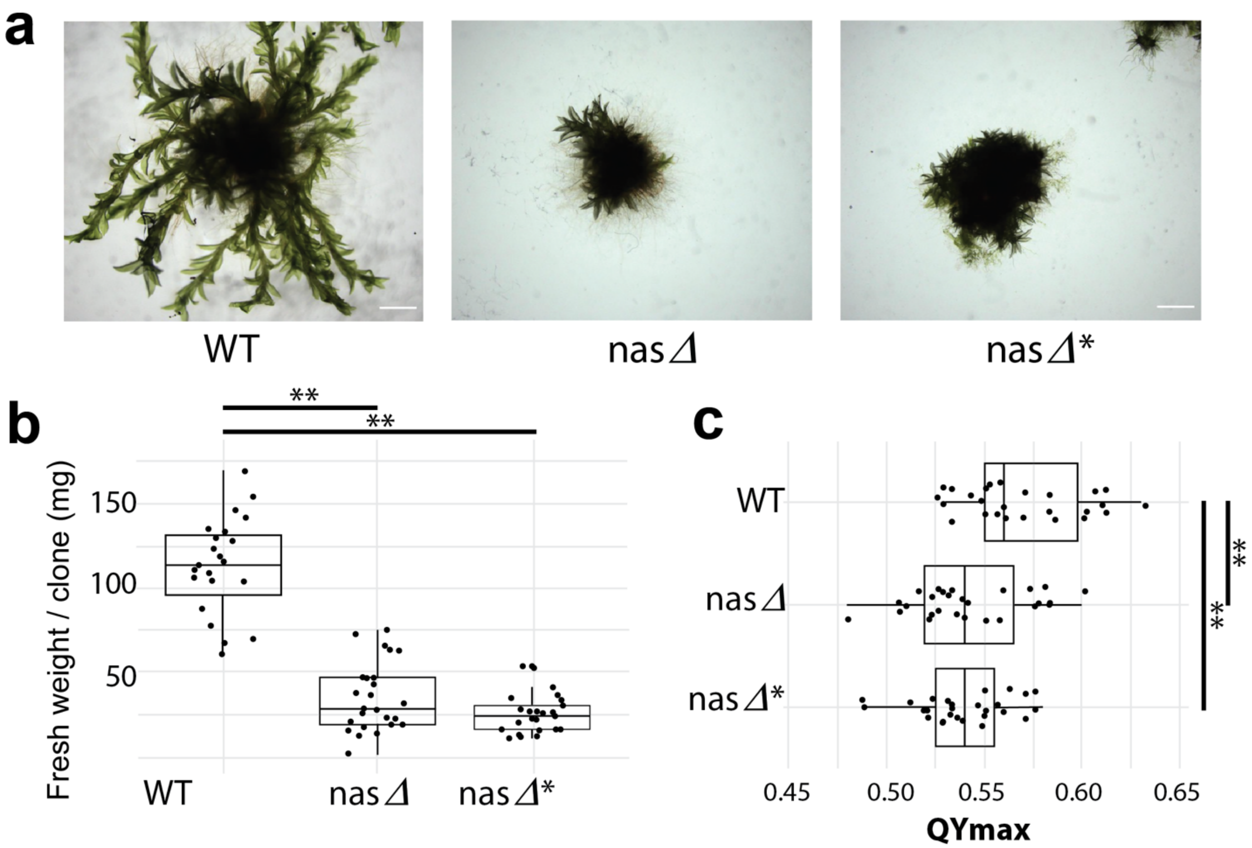

Supplementary Fig. 10 | Reduced fitness in *Physcomitrium patens* deleted for the NAS gene. **a,** Reduced growth and gametophore formation in *Physcomitrium patens nas* mutants. Mosses were grown on KNOP medium plus microelements and iron-citrate (20 µM) for 7 weeks, under standard conditions. WT wild type; *nasΔ2* and *nasΔ16* represent two independent *nas* mutant strains. Scale bar = 1 mm. **b,** Quantification of the growth defect. Bars graphs represent the means +/- SD. Measurements were conducted on distinct samples from 25 clones for each genotype (n=25) and statistical analysis performed using one-way Kruskal-Wallis/Tukey HSD test **p<0.01). Differences in growth between the two *nas* strains are insignificant (p=0.43). **c**, Maximum photosynthetic (QYmax=Fv/Fm) capacity of photosystem II (PSII) in wild type and *nas* deletion *P. patens* mutants. Differences in photosynthesis between the two *nas* strains are insignificant (p=0.89). Statistical analysis and clones are as in b; n=25 per genotype.

References

1. Husnik, F., McCutcheon, J. P. Functional horizontal gene transfer from bacteria to eukaryotes. *Nat. Rev. Microbiol.* 16, 67–79, doi: 10.1038/nrmicro.2017.137 (2018).
2. Bechteler, J. et al. Comprehensive phylogenomic time tree of bryophytes reveals deep relationships and uncovers gene incongruences in the last 500 million years of diversification. *Am. J. Bot.* **110**, e16249, doi:10.1002/ajb2.16249 (2023).
3. Goffinet, B., Buck, W.R., Shaw, A. J. Morphology and classification of the Bryophyta. In: *Bryophyte Biology* (eds Goffinet, B., Shaw, A. J.). 2nd Edition. Cambridge University Press (2009).
4. Carpenter, E. J. *et al.* Access to RNA-sequencing data from 1,173 plant species: The 1000 Plant transcriptomes initiative (1KP). *Gigascience* **8**, giz126, doi:10.1093/gigascience/giz126 (2019).
5. Kristensen, I. & Larsen, P. O. Azetidine-2-carboxylic acid derivatives from seeds of Fagus silvatica L. and a revised structure for nicotianamine. *Phytochemistry* **13**, 2791–2798 doi:10.1016/0031-9422(74)80243-8 (1974).
