# Supplementary material for: Multiple independent acquisitions of a metallophore-synthesis gene by plants through horizontal microbial gene transfer
Source: Nat Commun. 2025 Sep 22;16:8339. doi: 10.1038/s41467-025-61162-w (PMC12454661; doi:10.1038/s41467-025-61162-w)
Supplement: Supplementary file 4 — Description of Additional Supplementary Files [file 41467_2025_61162_MOESM4_ESM.pdf]

Description of Additional Supplementary Files

Supplementary Data 1

Description: List of bryophyte species for which genomes were screened for the presence of a NAS homolog, with reference to the original publication of the genomes, indication on the presence or absence of a NAS homolog, NCBI genome accession number of the genome and the genbank accession number of the NAS homolog.
