# Supplementary material for: Multiple independent acquisitions of a metallophore-synthesis gene by plants through horizontal microbial gene transfer
Source: Nat Commun. 2025 Sep 22;16:8339. doi: 10.1038/s41467-025-61162-w (PMC12454661; doi:10.1038/s41467-025-61162-w)
Supplement: Supplementary file 5 — Supplementary Data 1 [file 41467_2025_61162_MOESM5_ESM.pdf]

Supplementary Data

Multiple independent acquisitions of a metallophore-synthesis gene by plants through horizontal microbial gene transfer.

L. Dirick[1, @*], Y. Liu[2, 3, @], S. Dong[2], J. Yu[3], L. Ouerdane[4], Storti M.[5], A. Alboresi[5], C. Curie[1] & B. Goffinet[6*]

[1]IPSIM, Université Montpellier, CNRS, INRAE, Institut Agro, Montpellier, F-34060 France. [2]Key Laboratory of Southern Subtropical Plant Diversity, Fairy Lake Botanical Garden, Shenzhen & Chinese Academy of Sciences, Shenzhen, Guangdong, China. [3]BGI-Research, BGI-Wuhan, Wuhan 430074, China. [4]Université de Pau et des Pays de l'Adour, e2s UPPA, CNRS, IPREM-UMR5254, Hélioparc, 2, Av. Pr. Angot, 64053 Pau, France. [5]Dipartimento di Biologia, Università di Padova, Via Ugo Bassi 58b 35131, Padova, Italia. [6]Department of Ecology and Evolutionary Biology, 75 NorthEagleville road, University of Connecticut, Storrs CT, 06269-3043, USA. E-mail: leon.dirick@cnrs.fr, bernard.goffinet@uconn.edu

Supplementary Data 1 | List of bryophyte species for which genomes were screened for the presence of a NAS homolog, with reference to the original publication of the genomes, indication on the presence or absence of a NAS homolog, NCBI genome accession number of the genome and the genbank accession number of the NAS homolog**.**

Group	Species	DNA# / reference	NAS Homolog	NCBI Genome Acc.	NAS GenBank Acc.
hornwort	*Anthoceros agrestis*	Li et al. 2020	-	PRJNA574453	
hornwort	*Anthoceros angustus*	Zhang et al., 2020	-	PRJNA543716	
hornwort	*Anthoceros punctatus*	Li et al., 2020	-	PRJNA574453	
hornwort	*Folioceros fuciformis*	639, Dong et al., 2025	-	GCA_049999805.1	
hornwort	*Notothylas yunnanensis*	899, Dong et al., 2025	-	GCA_049999785.1	
hornwort	*Phaeoceros laevis*	1032, Dong et al., 2025	-	GCA_049999925.1	
hornwort	*Phaeoceros laevis*	902, Dong et al., 2025	-	GCA_049999765.1	
liverwort	*Acrobolbus urvilleanus*	626, Dong et al., 2025	-	GCA_049999545.1	

liverwort	*Acrolejeunea sandvicensis*	888, Dong et al., 2025	-	GCA_050084415.1	
liverwort	*Bazzania tridens*	1026, Dong et al., 2025	-	GCA_050001695.1	
liverwort	*Blasia pusilla*	890, Dong et al., 2025	+	GCA_050084515.1	PV665650
liverwort	*Blasia pusilla*	349, Dong et al., 2025	+	GCA_049999905.1	PV665651
liverwort	*Conocephalum conicum*	664, Dong et al., 2025	-	GCA_050084475.1	
liverwort	*Cyathodium cavernarum*	908, Dong et al., 2025	-	GCA_050084455.1	
liverwort	*Fossombronia cristula*	889, Dong et al., 2025	-	GCA_050001065.1	
liverwort	*Frullania moniliata*	957, Dong et al., 2025	-	GCA_050001525.1	
liverwort	*Gackstroemia magellanica*	604, Dong et al., 2025	-	GCA_049999665.1	
liverwort	*Haplomitrium mnioides*	302, Dong et al., 2025	-	GCA_049999725.1	
liverwort	*Herbertus kurzii*	906, Dong et al., 2025	-	GCA_049999405.1	
liverwort	*Lepidozia reptans*	961, Dong et al., 2025	-	GCA_050001415.1	
liverwort	*Lunularia cruciata*	231, Dong et al., 2025	-	GCA_049999865.1	
liverwort	*Marchantia polymorpha*	Bowman et al., 2017	-	GCA_037833965.1	
liverwort	*Mesoptychia sp.*	623, Dong et al., 2025	-	GCA_049999605.1	
liverwort	*Metzgeria furcata*	368, Dong et al., 2025	-	GCA_050001055.1	
liverwort	*Metzgeria hamata*	905, Dong et al., 2025	-	GCA_049999825.1	
liverwort	*Noteroclada confluens*	625, Dong et al., 2025	-	GCA_049999745.1	
liverwort	*Odontoschisma sphagni*	343, Dong et al., 2025	-	GCA_049999585.1	
liverwort	*Pallavicinia ambigua*	912, Dong et al., 2025	-	GCA_050000995.1	
liverwort	*Plagiochasma appendiculatum*	952, Dong et al., 2025	-	GCA_050084495.1	
liverwort	*Plagiochila semidecurrens*	369, Dong et al., 2025	-	GCA_049999485.1	
liverwort	*Plagiochila sp.*	621, Dong et al., 2025	-	GCA_050001775.1	
liverwort	*Pleurozia purpurea*	891, Dong et al., 2025	-	GCA_049999845.1	
liverwort	*Plicanthus hirtellus*	963, Dong et al., 2025	-	GCA_049999505.1	
liverwort	*Porella caespitans var. nipponica*	1017, Dong et al., 2025	-	GCA_049999625.1	
liverwort	*Porella chinensis*	1025, Dong et al., 2025	-	GCA_049999645.1	
liverwort	*Porella platyphylla*	630, Dong et al., 2025	-	GCA_050001495.1	

liverwort	*Ptilidium pulcherrimum*	225, Dong et al., 2025	-	GCA_049999685.1	
liverwort	*Ricciocarpos natans*	909, Dong et al., 2025	-	GCA_050084435.1	
liverwort	*Scapania ferruginea*	359, Dong et al., 2025	-	GCA_050001655.1	
liverwort	*Solenostoma erectum*	955, Dong et al., 2025	-	GCA_050084395.1	
liverwort	*Sphaerocarpos donnellii*	M158, Dong et al., 2025	-	GCA_049999885.1	
liverwort	*Tetralophozia filiformis*	965, Dong et al., 2025	-	GCA_050000895.1	
liverwort	*Treubia lacunosa*	1016, Dong et al., 2025	-	GCA_049999705.1	
liverwort	*Tritomaria exsectiformis*	966, Dong et al., 2025	-	GCA_049999525.1	
liverwort	*Vetaforma dusenii*	620, Dong et al., 2025	-	GCA_049999425.1	
moss	*Aerobryopsis subdivergens*	668, Dong et al., 2025	+	GCA_050083985.1	PV634254, PV634255
moss	*Andreaea rupestris*	658, Dong et al., 2025	-	GCA_050001315.1	
moss	*Andreaea wilsonii*	1012, Dong et al., 2025	-	GCA_050001515.1	
moss	*Andreaeobryum macrosporum*	350, Dong et al., 2025	-	GCA_049999365.1	
moss	*Archidium alternifolium*	1127, Dong et al., 2025	+	GCA_050084235.1	PV634250
moss	*Atrichum angustatum*	1015, Dong et al., 2025	+	GCA_050084355.1	PV634249
moss	*Aulacomnium palustre*	324, Dong et al., 2025	+	GCA_050002695.1	PV634248
moss	*Aulacomnium turgidum*	783, Zeng et al., 2025	+	GCA_048933245.1	PV634247
moss	*Bartramia mossmaniana*	613, Dong et al., 2025	+	GCA_050002155.1	PV634246
moss	*Brachythecium laetum*	336, Dong et al., 2025	+	GCA_050003315.1	PV634245
moss	*Bryhnia novae-angliae*	342, Dong et al., 2025	+	GCA_050002955.1	PV634244
moss	*Bryoandersonia illecebra*	337, Dong et al., 2025	+	GCA_050002995.1	PV634243
moss	*Bryoxiphium norvegicum*	911, Dong et al., 2025	+	GCA_050084295.1	PV634242
moss	*Bryum knowltonii*	782, Dong et al., 2025	+		PV634240, PV634241
moss	*Bucklandiella nitidula*	958, Dong et al., 2025	+	GCA_050001675	PV634239
moss	*Buxbaumia aphylla*	646, Dong et al., 2025	-	GCA_050001715.1	
moss	*Calliergon cordifolium*	1036, Dong et al., 2025	+	GCA_049999265.1	PV634238
moss	*Calliergonella curvifolia*	Yu et al., 2022	+	CNP0002064, CNGB Sequence Archive (CNSA)	PV634237
moss	*Calohypnum plumiforme*	Mao et al., 2020	+	PRJCA001833, Genome Sequence Archive (GSA)	

moss	*Catagonium nitens*	600, Dong et al., 2025	+	GCA_050003415.1	PV634228
moss	*Catagonium nitens*	617, Dong et al., 2025	+	GCA_050002935.1	PV634235
moss	*Catagonium nitens*	618, Dong et al., 2025	+	GCA_050002855.1	PV665652
moss	*Ceratodon purpureus*	M758, Carey et al., 2021	+	JACMSA000000000	
moss	*Ceratodon purpureus*	KC19, Carey et al., 2021	+	JACMSB000000000	
moss	*Chorisodontium aciphyllum*	655, Dong et al., 2025	+		PV634233
moss	*Chorisodontium aciphyllum*	616, Dong et al., 2025	+	GCA_050002275.1	PV634234
moss	*Climacium americanum*	322, Dong et al., 2025	+	GCA_050003075.1	PV634232
moss	*Cratoneuron filicinum*	960B, Dong et al., 2025	+	GCA_050003555.1	PV634230, PV634231
moss	*Dicranoloma chilense*	612, Dong et al., 2025	+	GCA_050002295.1	PV634229
moss	*Diphyscium fulvifolium*	910, Dong et al., 2025	-	GCA_050084335.1	
moss	*Distichophyllum collenchymatosum*	969, Dong et al., 2025	-	GCA_050002395.1	
moss	*Elmeriobryum philippinense*	954, Dong et al., 2025	+	GCA_050003195.1	PV634226
moss	*Encalypta ciliata*	1125, Dong et al., 2025	+	GCA_050002015.1	PV665653
moss	*Entodon concinnus*	964, Dong et al., 2025	+	GCA_050083955.1	PV634236
moss	*Entodon seductrix*	Yu et al., 2022	+	CNP0002064, CNGB Sequence Archive (CNSA)	PV634225
moss	*Fissidens javanicus*	898, Dong et al., 2025	+	GCA_050084195.1	PV634224
moss	*Fontinalis sullivantii*	334, Dong et al., 2025	+	GCA_050002785.1	PV665654
moss	*Funaria hygrometrica*	301, Kirbis et al., 2025	+	CNP0002793, CNGB Sequence Archive (CNSA)	PV634223
moss	*Gigaspermum repens*	1151, Dong et al., 2025	-	GCA_049999385.1	
moss	*Grimmia obtusifolia*	1123, Dong et al., 2025	+	GCA_050084275.1	PV634222
moss	*Hedwigia ciliata*	1009, Dong et al., 2025	+	GCA_050084075.1	PV634221
moss	*Hedwigia ciliata*	329, Dong et al., 2025	+	GCA_050002575.1	PV634220
moss	*Homaliodendron scalpellifolium*	1024, Dong et al., 2025	+	GCA_049999225.1	PV634219
moss	*Hydrogonium amplexifolium*	967, Dong et al., 2025	+	GCA_050084095.1	PV634218
moss	*Hygroamblystegium varium*	344, Dong et al., 2025	+	GCA_050003535.1	PV634252, PV634253
moss	*Hygrohypnum luridum*	960A, Dong et al., 2025	+	GCA_050003155.1	PV634217
moss	*Hypopterygium elatum*	1020, Dong et al., 2025	+	GCA_050002555.1	PV634216

moss	*Hypopterygium flavolimbatum*	893, Dong et al., 2025	+	GCA_050002675.1	PV634214, PV634215
moss	*Kindbergia praelonga*	628, Dong et al., 2025	+	GCA_049999245.1	PV634212, PV634213
moss	*Leptotheca gaudichaudii*	615, Dong et al., 2025	+	GCA_050002715.1	PV634211
moss	*Lepyrodon lagurus*	601, Dong et al., 2025	+	GCA_050002835.1	PV634210
moss	*Leucobryum albidum*	323, Dong et al., 2025	+	GCA_049999345.1	PV634209
moss	*Leucobryum bowringii*	671, Dong et al., 2025	+	GCA_050084215.1	PV634208
moss	*Mnium hornum*	338, Dong et al., 2025	+	GCA_050003115.1	PV634207
moss	*Orthodicranum fulvum*	335, Dong et al., 2025	+	GCA_049999305.1	PV634227
moss	*Orthotrichum anomalum*	795, Dong et al., 2025	+	GCA_050084045.1	PV634206
moss	*Paraleucobryum enerve*	1124, Dong et al., 2025	+	GCA_050084115.1	PV634204, PV634205
moss	*Philonotis turneriana*	665, Dong et al., 2025	+	GCA_050084035.1	PV634203
moss	*Physcomitrium patens*	Lang et al., 2018	+	GCF_000002425.5	
moss	*Plagiomnium ciliare*	341, Dong et al., 2025	+	GCA_049999325.1	PV634202
moss	*Pleuridium subulatum*	1013, Dong et al., 2025	+	GCA_050001895.1	PV634201
moss	*Pogonatum microstomum*	956, Dong et al., 2025	+	GCA_050001535.1	PV634200
moss	*Pogonatum subfuscatum*	1092, Dong et al., 2025	+	GCA_050002065.1	PV634199
moss	*Polytrichadelphus magellanicus*	614, Dong et al., 2025	+	GCA_050002135.1	PV634198
moss	*Polytrichastrum alpinum*	660, Zeng et al., 2025	+	GCA_048933195.1	PV634197
moss	*Polytrichastrum ohioense*	1035, Dong et al., 2025	+	GCA_050002035.1	PV634196
moss	*Polytrichum commune*	1089, Dong et al., 2025	+	GCA_050001815.1	PV634195
moss	*Polytrichum strictum*	607, Dong et al., 2025	+	GCA_050001795.1	PV634194
moss	*Pseudanomodon attenuatus*	340, Dong et al., 2025	+	GCA_050003055.1	PV634251
moss	*Pseudobryum cinclidioides*	325, Dong et al., 2025	+	GCA_049999285.1	PV634193
moss	*Ptychomitrium wilsonii*	670, Dong et al., 2025	+	GCA_050084255.1	PV634192
moss	*Ptychomnion cygnisetum*	606, Dong et al., 2025	+	GCA_050002605.1	PV634191
moss	*Pyrrhobryum spiniforme*	667, Dong et al., 2025	+	GCA_050084015.1	PV665655
moss	*Rhytidiadelphus subpinnatus*	627, Dong et al., 2025	+	GCA_050003335.1	PV634190
moss	*Sanionia uncinata*	654, Dong et al., 2025	+		PV634189

moss	*Schistidium cupulare*	608, Dong et al., 2025	+	GCA_050001915.1	PV634188
moss	*Sphagnum girgensohnii*	904, Dong et al., 2025	-	GCA_050001455.1	
moss	*Sphagnum palustre*	644, Dong et al., 2025	-	GCA_050001735.1	
moss	*Splachnum ampullaceum*	1120, Dong et al., 2025	+	GCA_050002815.1	PV634186, PV634187
moss	*Syntrichia caninervis*	Silva et al., 2021	+	GCA_016097705.1	
moss	*Syntrichia ruralis*	787, Dong et al., 2025	+		PV634184, PV634185
moss	*Syntrichia ruralis*	1122, Dong et al., 2025	+	GCA_050002175.1	PV634183
moss	*Takakia lepidozioides*	Hu et al., 2023	-	GCA_030704615.1	
moss	*Tetraphis pellucida*	1011, Dong et al., 2025	+	GCA_050084375.1	PV634182
moss	*Tetraphis pellucida*	328, Dong et al., 2025	+	GCA_050001755.1	PV634181
moss	*Thamnobryum sandei*	962, Dong et al., 2025	+	GCA_050003295.1	PV634179, PV634180
moss	*Timmia megapolitana*	1010, Dong et al., 2025	+	GCA_050084315.1	PV634178
moss	*Ulota hutchinsiae*	326, Dong et al., 2025	+	GCA_050002435.1	PV634177

References

Bowman, J. L. et al. Insights into land plant evolution garnered from the Marchantia polymorpha genome. *Cell* **171**, 287–304, doi: 10.1016/j.cell.2017.09.030 (2017).

Dong, S. et al. Bryophytes hold a larger gene family space than vascular plants. *Nat. Gen.* (2025).

Hu R., X. *et al.* Adaptive evolution of the enigmatic Takakia now facing climate change in Tibet *Cell* 186: 3558–3576, doi: 10.1016/j.cell.2023.07.003 (2023).

Carey, S. B. et al. Gene-rich UV sex chromosomes harbor conserved regulators of sexual development. *Sci. Adv.* **7**: eabh2488, doi: 10.1126/sciadv.abh2488 (2021).

Kirbis, A. et al. Comparative analysis of the Funaria hygrometrica genome suggests greater collinearity in mosses than in seed plants. *Commun. Biol.* **8**, 330, doi: 10.1038/s42003-025-07749-x (2025).

Lang, D. et al. The Physcomitrella patens chromosome-scale assembly reveals moss genome structure and evolution. *Plant J.* **93**, 515–533, doi: 10.1111/tpj.13801 (2018).

Li, F. W. et al. Anthoceros genomes illuminate the origin of land plants and the unique biology of hornworts. *Nat. Plants* **6**, 259–272, doi: 10.1038/s41477-020-0618-2 (2020).

Mao, L. et al. Genomic evidence for convergent evolution of gene clusters for momilactone biosynthesis in land plants. *Proc. Natl. Acad. Sci. U S A* **117**, 12472–12480, doi: 10.1073/pnas.1914373117 (2020).

Silva, A. T. et al. To dry perchance to live: Insights from the genome of the desiccation-tolerant biocrust moss Syntrichia caninervis. *Plant J.* **105**, 1339–1356, doi: 10.1111/tpj.15116 (2021).

Yu, J. et al. Chromosome-level genome assemblies of two Hypnales (mosses) reveal high intergeneric synteny. *Genome Biol. Evol.* **14**, evac020, doi: 10.1093/gbe/evac020 (2022).

Zhang, J. et al. The hornwort genome and early land plant evolution. *Nat. Plants* **6**, 107–118, doi:10.1038/s41477-019-0588-4 (2020).
