# Supplementary material for: Multiple independent acquisitions of a metallophore-synthesis gene by plants through horizontal microbial gene transfer
Source: Nat Commun. 2025 Sep 22;16:8339. doi: 10.1038/s41467-025-61162-w (PMC12454661; doi:10.1038/s41467-025-61162-w)
Supplement: Supplementary file 2 — Transparent Peer Review file [file 41467_2025_61162_MOESM2_ESM.pdf]

nature portfolio

Peer Review File

Multiple independent acquisitions of a metallophore-synthesis gene by plants through horizontal microbial gene transfer

Corresponding Author: Professor Bernard Goffinet

This file contains all reviewer reports in order by version, followed by all author rebuttals in order by version.

Version 0:

Reviewer comments:

Reviewer #1

(Remarks to the Author)
This is a very interesting manuscript, reporting a remarkable case of recurrent HGT of nicotiamine synthase to mosses. I was skeptical at first but the authors' data is convincing - HGT is the most logical explanation. I want to highlight that the authors went beyond phylogenetic analyses and incorporated gene functional studies as well as mass-spec to make their case.

(1) Recurrent HGT of the same gene family from bacteria to plants was actually reported previously as part of the Azolla/Salvinia genome paper (Li et al 2018 Nature Plants). See the discussion on squalene-hopene cyclase and also Supplementary Fig. 12. In this sense the statement on line 47-49 is not accurate.

(2) This study relies heavily on the unpublished moss genomes. I'm not sure what's the policy for Nature Communications, but I'd like to know more about these genomes. Were they derived from axenic cultures? What are the assembly and annotation stats? BUSCO completeness? Where and when will the data be publicly available?

(3) The absence of NAS in Distichophyllum could also be explained by incomplete genome assembly or annotation error. Secondary loss may not be the only explanation.

(4) To rule out contamination, the authors examined the flanking genes of moss NAS and determined that they are "unambiguous plant genes". However it is hard to tell from Supp Fig. 4 alone that these are plant genes or not – all it says is that most genes have best hits to Physco or Marchantia genes, which can still be of bacterial/fungal origins. I'd suggest carrying out phylogenetic analyses on the flanking genes as well.

(5) A key evidence supporting moss NAS being bona fide plant genes came from introns, but surprisingly this wasn't mentioned in the text at all and only appeared in Fig. 2. The authors could strengthen their augment by having an in-depth discussion on introns.

(6) Gene tree/species tree congruence could be yet another way to rule out contamination. It seems to be the case from looking at the supplementary figures, but it was not discussed in the manuscript.

(7) Fig. 3 – That is not how Blasia should look like!

Reviewer #2

(Remarks to the Author)
The manuscript submitted by Dirick et al. provides some evidences of multiple cross-kingdom HGTs of nicotiamine synthase gene in land plants. I would like first to say that the results, as for the HGTs detection, are convincing, despite the fact that only one (phylogenetic incongruence, PI) among the three criteria commonly used for the detection of HGTs has been examined. Among the other two, the patchy distribution (PD) is somehow also met, although the authors do not really mention it, while the third one, the high similarity (HS) is not mentioned at all. The main reason for this is that the HGTs

described here are very ancient and that among the three criteria, only the PI remains really useful.
I also found the manuscrit very well written and focused. I, however have two problems with it which, in my opinion, prevent its publication in NC as it stands :

- Orthology and gene replacement :
Throughout the manuscript, the authors use the term "orthologs", which is very confusing, unless I haven't fully understand their result : orthologs are homologs (with common ancestors) that remains at the same genomic location after an evolutionary split (eg speciation). orthology is sometimes invoked when the function of the gene is conserved, although the homolog is located at a different locus. In such case, one should maybe better refer to functional homologs, but this is also misleading. Why this remark ? Physcomitrella patens is known to integrate foreign DNA through homologous recombination (HR). Throughout the manuscript, when the authors invoke orthology, one wonders whether there are bona fide orthologous relationships among the horizontally transferred NAS, with conserved synteny, in particular whether the "replacement" that the authors mentioned in several instances has been obtained through HR or not, which is very important from an evolutionary perspective ?: if this is the case, frequent HGTs followed by HR could easily explain the propensity of bryophytes to diversify their gene space. If this is not, replacement could only be the rsult of a gain (through HGT) followed by a loss, but in this case, why isn't there a trace of the process, for instance in the form of pseudogenized NAS ? The authors mention that they rule out the possibility of contamination by verifyinig that the genes flanking the NAS are bona fide plant genes. Are these the same in the different lineages ?

- flowering plants.
If the authors ambition to publish in a high profile journal like NC, I think they should extend their analysis to the angiosperms and (to a lesser extent) the gymnosperms genomes available. I understand that some of the co-authors have an excellent expertise in Arabidopsis NAS, but the lack of results on other angiosperems lineages is a big miss. Are there as many HGTs in flowerinig plants (for which HR is not possible) than in bryophytes ?

Reviewer #3

(Remarks to the Author)
This is a very well written and conceived, highly original manuscript that considerably extend understanding of horizontal transfer of microbial genes in land plants by focusing on early diverging lineages (bryophytes). By broadening the search of the metallophore-synthesis NAS gene in mosses beyond the model species Physcomitrium patens, and including more than hundred new bryophyte genomes, the authors are able to show, for the first time, that the NAS genes have been acquired independently multiple times and from multiple microbial donors during the diversification of land plants. Thus, their findings challenge the current view of homology of microbial genes in plants, demonstrating instead that microbial gene exchange is much more dynamic than assumed. These are very important findings with the potential to provide a new layer of understanding to how plants conquered and diversified on land.
Although I am not an expert in all of the techniques, the methods appear sound, are clearly presented and easy to follow, and all the conclusions seem to be strongly backed by the results, which are also extremely well presented.
Overall, the manuscript was a pleasure to read and review. I found it intriguing that, within bryophytes, NAS genes are present only in mosses, which altogether lack mycorrhizal-like associations (I would be cautious of reports of these in Takakia), and only in one liverwort, Blasia pusilla, in the Blasiales, which stands out amongst liverworts not only for their symbioses with cyanobacteria but also as the only early divergent lineage that lack mycorrhizal-like associations. Perhaps the authors could also consider this latter point in their discussion.
Other than this, I have no suggestions as to make changes to the paper, which I find excellent. I was only able to spot a few minor typos that need correcting (e.g. Line 146: absences; Line 175: addition; Line 247: analyses were implemented; Line 314: 21oC); although the reference list could benefit from some careful editing.

Version 2:

Reviewer comments:

Reviewer #1

(Remarks to the Author)
The authors have addressed most of my previous comments nicely. The statement “as in the case of the squalene synthase gene, transferred independently from cyanobacteria to bryophytes and to ferns.” Is not correct. It should be “squalene-hopene cyclase” and the donors of the bryophyte copies are not Cyanobacteria.

In the response to reviewers’ document, what is Blasia pellucida?

I still have concerns about data availability, but I will leave this to the editor to coordinate. When this paper is published, the genome data should be released as well.

In any case, very interesting finding!! Congrats!

Reviewer #2

(Remarks to the Author)
The authors have done a good job with addressing the points that I have raised, however they haven't revised the manuscript accordingly (see below).

"Response: We assume that the reviewer refers to the three criteria by Leger et al. (BioEssays 2018, 40, e1700242) and referred to by Aubin et al. (cited in our manuscript)"

The three criteria were initially published by Wallau et al., GBE 2012.

"While we do not report on sequence similarity per se, we do reflect patterns of similarity of plant NAS genes to various microbial NAS genes, via our phylogenetic reconstructions (Figs. 1 & S1). Our conclusion of independent transfers is explicitly based on the phylogenetic incongruence (gene versus species trees: Figs. 1, S1, S2 & S3) and patchy distribution in phylogenies (Figs. 1 & S1)."

This reply is OK to me but the text has not been appended accordingly.

"Regarding homologous recombination: the replacement that we hypothesize of the FunNAS by a HypNAS, pertains to genes occurring at different genomic location (FunNAS integrated on Physcomitrium chr 8) is at a different locus than the HypNAS,
which is the chromosome equivalent to Pp Chr18/21, and these chromosomes are not homeologs arising from an ancient whole genome duplication (see Lang et al., The Plant Journal, 2018, 93, 515–533). If HR is involved in the GT events, it might be through
micro-homologies (for instance of repeated sequences/transposition sites), not by replacement of a NAS gene by a second one through HR at the same locus. In the
example we describe (replacement of FunNAS by HypNAS), the most likely scenario is the integration of the HypNAS in the ancestor to the Bryidae (from Bryales to Hypnales in our phylogeny), bearing an FunNAS copy, followed by gene loss of the FunNAS copy."

Why is this not mentioned in the new version ?

"Regarding pseudogene: The loss of the FunNAS would likely date back to 300–360 million years, based on the chronograms29 and hence be too ancient for a pseudogene to be retained."

I am ok with this. But why is this not mentioned in the new version ?

Open Access This Peer Review File is licensed under a Creative Commons Attribution 4.0 International License, which permits use, sharing, adaptation, distribution and reproduction in any medium or format, as long as you give appropriate credit to the original author(s) and the source, provide a link to the Creative Commons license, and indicate if changes were made.

In cases where reviewers are anonymous, credit should be given to 'Anonymous Referee' and the source.
The images or other third party material in this Peer Review File are included in the article's Creative Commons license, unless indicated otherwise in a credit line to the material. If material is not included in the article's Creative Commons license and your intended use is not permitted by statutory regulation or exceeds the permitted use, you will need to obtain permission directly from the copyright holder.
To view a copy of this license, visit https://creativecommons.org/licenses/by/4.0/

Reviewer #1 (Remarks to the Author):

This is a very interesting manuscript, reporting a remarkable case of recurrent HGT of nicotiamine synthase to mosses. I was skeptical at first but the authors' data is convincing - HGT is the most logical explanation. I want to highlight that the authors went beyond phylogenetic analyses and incorporated gene functional studies as well as mass-spec to make their case.

(1) Recurrent HGT of the same gene family from bacteria to plants was actually reported previously as part of the Azolla/Salvinia genome paper (Li et al 2018 Nature Plants). See the discussion on squalene-hopene cyclase and also Supplementary Fig. 12. In this sense the statement on line 47-49 is not accurate.

Response: Thank you. This is now added in the last paragraph starting on line 237:
Reconstructing the evolution of genes of microbial origin during the diversification of plants within a comprehensive sampling of land plants revealed the potentially highly dynamic nature of such transfers from a suite of donors, and that transferred genes are more frequently becoming fixed, i.e., entering the germ line and integrated to benefit to plant, than might have been previously assumed or accepted [46 36]*, as in the case of the squalene synthase gene, transferred independently from cyanobacteria to bryophytes and to ferns*[23]*.*

(2) This study relies heavily on the unpublished moss genomes. I'm not sure what's the policy for Nature Communications, but I'd like to know more about these genomes. Were they derived from axenic cultures? What are the assembly and annotation stats? BUSCO completeness? Where and when will the data be publicly available?

Response: The novel genomic data for bryophytes upon which the current study is based are released as part of another study, which is providing the details of the quality of the assemblies. The genomes were extracted from wild populations. We have provided a summary statement on the origin and assembly of the genomes and provided BUSCO score for specific taxa in the discussion.

(3) The absence of NAS in *Distichophyllum* could also be explained by incomplete genome assembly or annotation error. Secondary loss may not be the only explanation.

Response: We have added a statement in the text on line 102:
Given the high quality of its genome assembly (i.e., a BUSCO score of 96.3% based on the Viridiplantae odb10 data set, the absence of the NAS gene is best explained by a secondary loss of the HypNAS (Fig. 2).

(4) To rule out contamination, the authors examined the flanking genes of moss NAS and determined that they are "unambiguous plant genes". However it is hard to tell from Supp Fig. 4 alone that these are plant genes or not – all it says is that most genes have best hits to Physco or Marchantia genes, which can still be of bacterial/fungal origins. I'd suggest carrying out phylogenetic analyses on the flanking genes as well.

Response: Among the genes flanking the NAS gene in bryophytes, Laminarase and MID1-C (FunNAS neighbors), NUP133 and RINGH2-ATL (HypNAS neighbors) and ETO1-LIKE (PolNAS neighbor, left), have no microbial homologs, and are only known from plants.

We performed fast phylogenetic analyses for the remainder, which have homologs among microbes: PWP2 (PolNAS neighbor, right), Luminal_B and von Willebrand Factor-A (TetNAS neighbors, left and right), PIF2 and SPINSTER (BlaNAS neighbors, left and right).

In all instances, phylogenetic inferences resolve the NAS flanking genes among plant homologs, and not with microbial sequences, as might be expected for a bona fide HGT in a plant genome (see below). Also, as demonstrated in the manuscript the gene trees of the FunNAS and HypNAs reflect the species tree and therefore support the hypothesis that the genes are indeed bryophyte orthologs in their respective clades. Phylogenetic gene trees for these loci are shown below. A blue asterisk marks the sequence of the NAS neighbor.

Luminal B

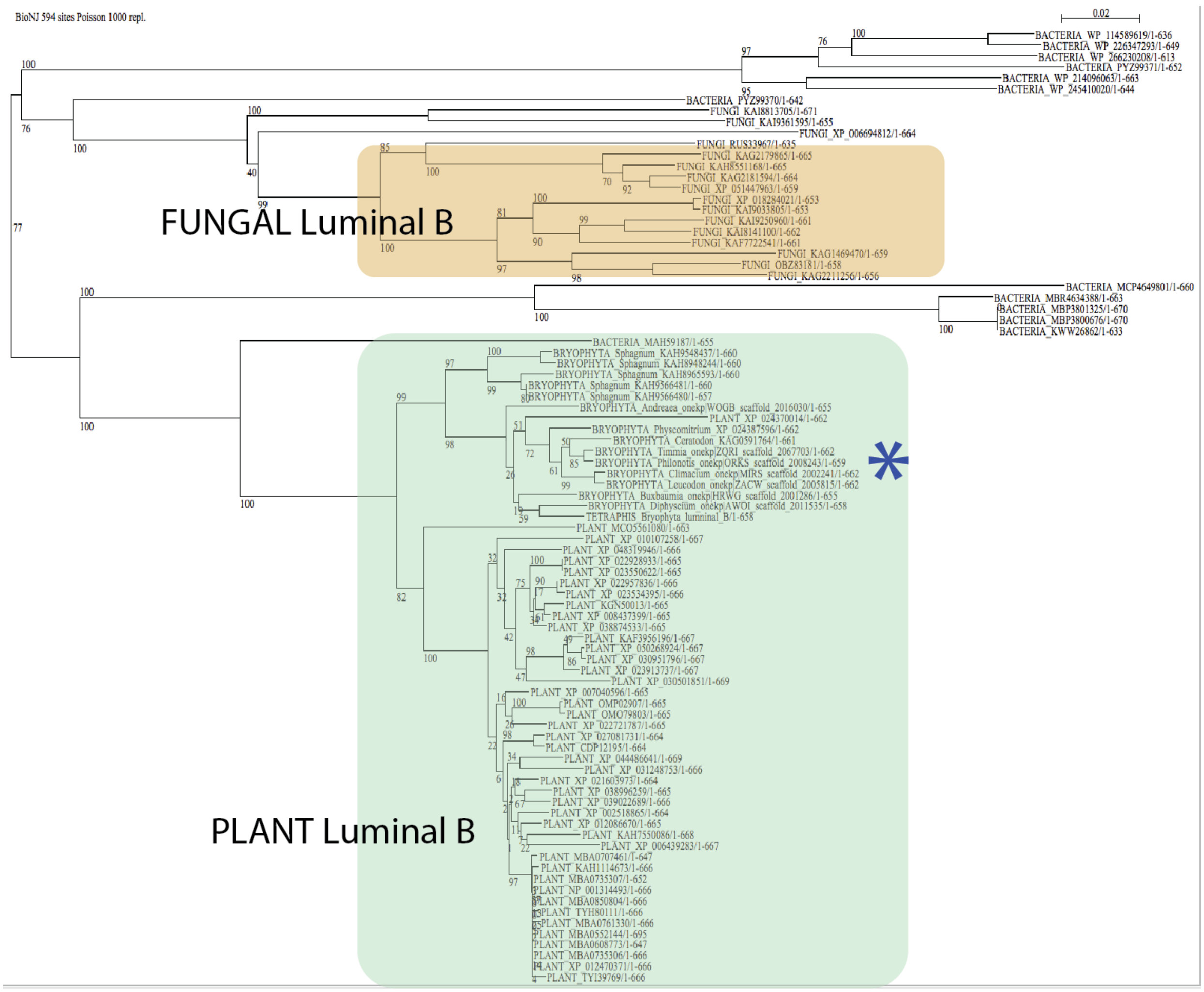

Gene tree for the Luminal B gene flanking the TetNAS gene in the moss *Tetraphis pellucida*, showing that plant Luminal B genes compose a monophyletic group comprising only plant Luminal B orthologs (green box), and all moss orthologs (star) share a unique common ancestor.

von_Willebrand_factor_A

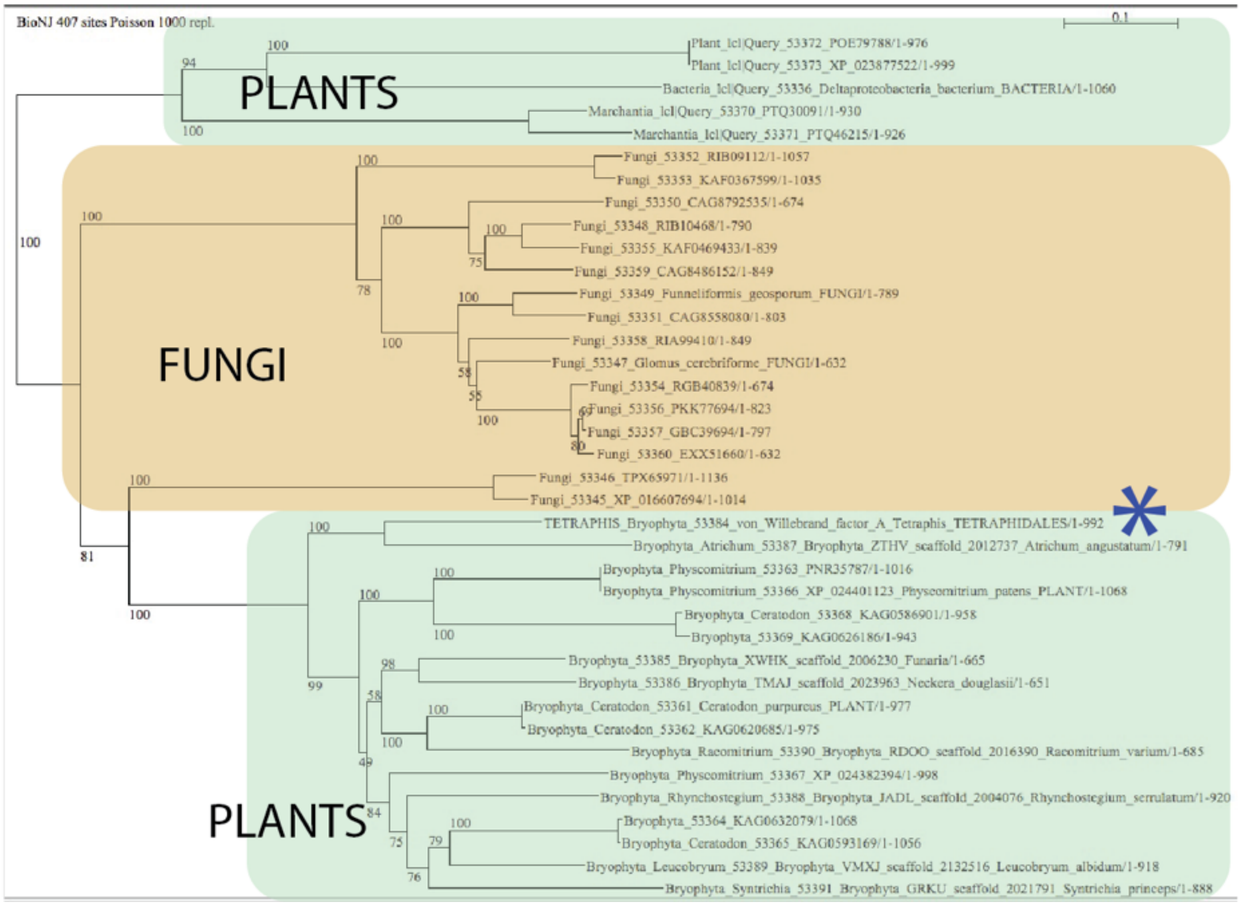

Unrooted gene tree for the von_Willebrand_factor_a gene flanking the TetNAS gene in the moss *Tetraphis pellucida*, showing that the *Tetraphis* ortholog (star) is nested among plant orthologs (green box) and sister to the ortholog of the moss of the Polytrichopsida (i.e., *Atrichum*).

Pif2

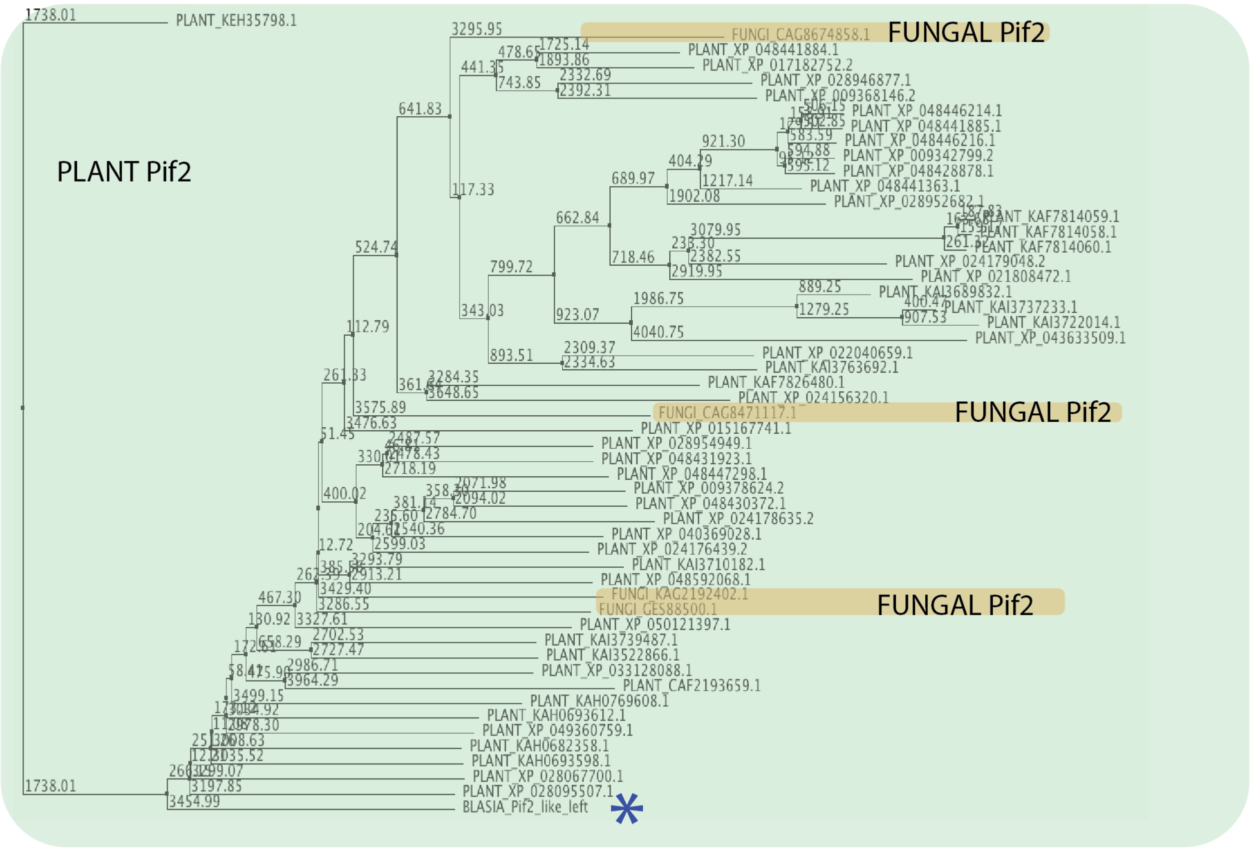

Unrooted gene tree for the Pif2 gene flanking the BlaNAS gene in the liverwort *Blasia pellucida*, showing that the *Blasia* ortholog (star) is nested between plant orthologs (green box).

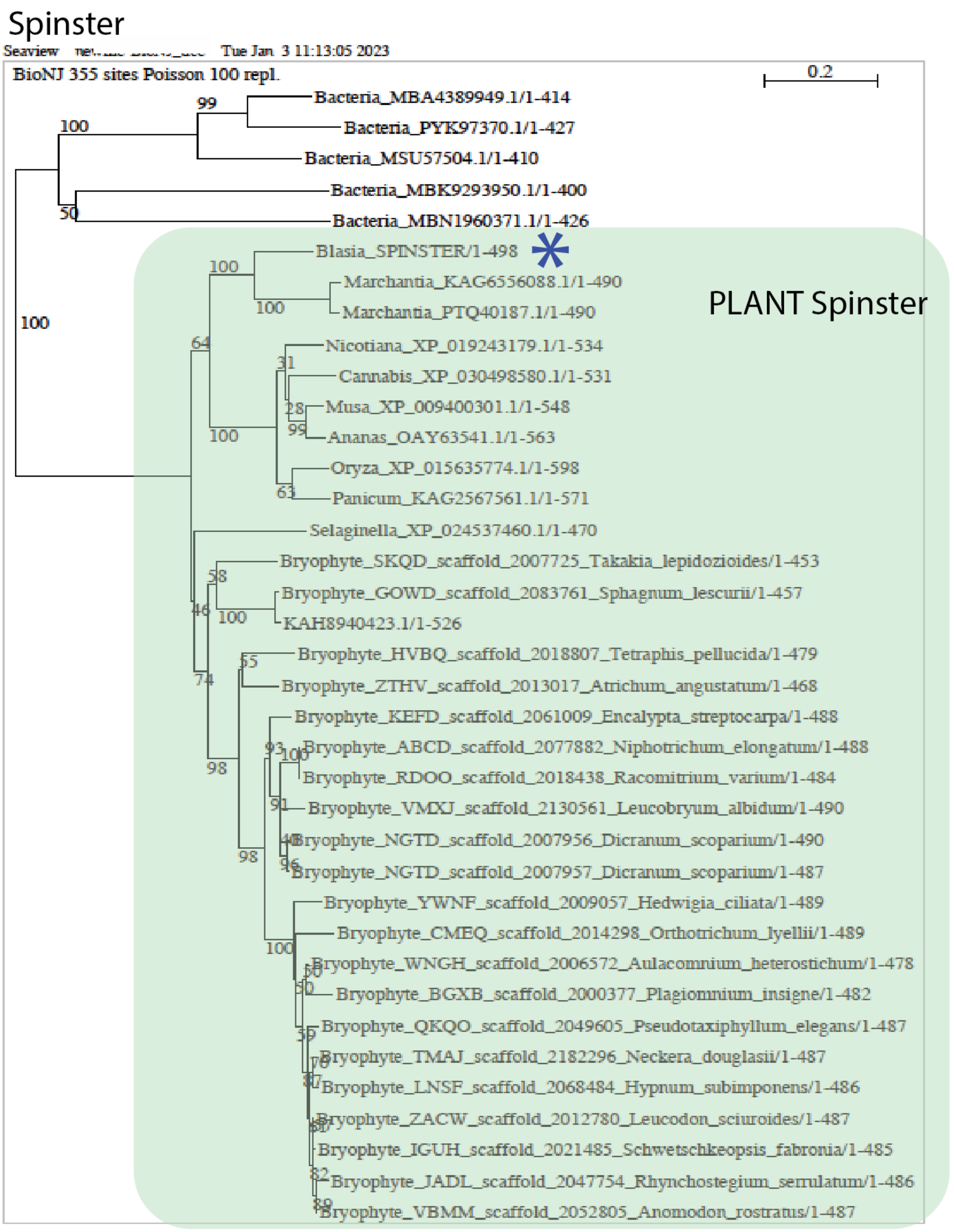

Unrooted gene tree for the Spinster gene flanking the BlaNAS gene in the liverwort *Blasia pellucida*, showing that the *Blasia* ortholog (star) is nested among plant orthologs (green box) and sister to the ortholog of the other liverwort *Marchantia*.

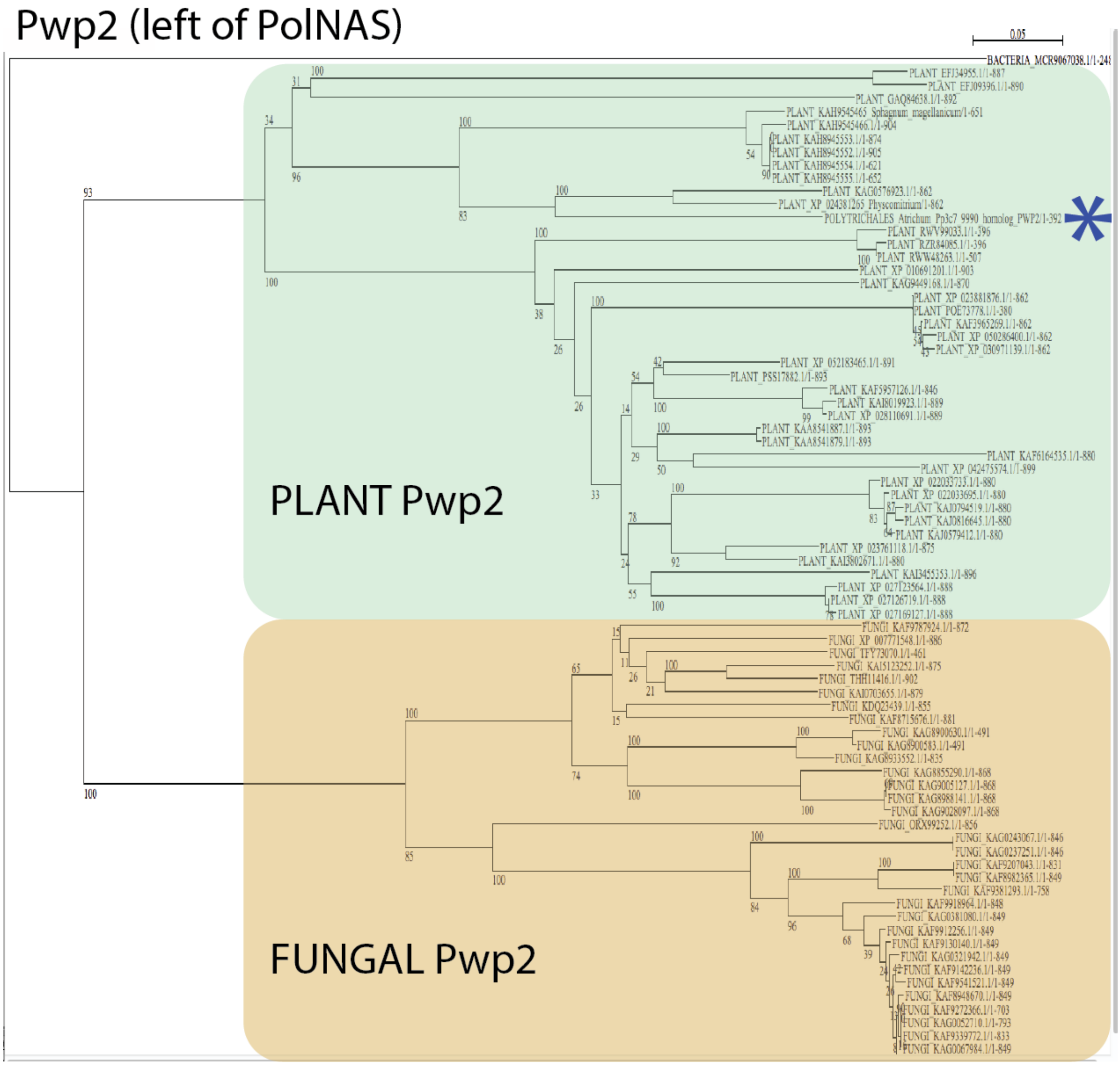

Gene tree for the Pwp2 gene flanking the PolNAS gene in the moss family Polytrichaceae, showing that the Pwp2 ortholog from *Atrichum* (star) representative of this family nested among plant orthologs (green box).

(5) A key evidence supporting moss NAS being bona fide plant genes came from introns, but surprisingly this wasn't mentioned in the text at all and only appeared in Fig. 2. The authors could strengthen their argument by having an in-depth discussion on introns.

Response: The NAS in plants either holds an intron or is intronless. In vascular plants NAS genes are intronless, with few exceptions, f.e. in Cucurbitaceae, with an intron likely acquired much after the original HGT (data not shown). In mosses, the TetNAS, PolNAS and HypNAS are intron free, except for the HypNAS in the Bryales, and only the FunNAS universally includes an intron. The pattern observed in mosses is not informative regarding the origin of their NAS gene, and we prefer not to speculate on the evolution of the NAS intron, as it does not inform or

nuance the pattern of proposed NAS acquisition by plants, as we present strong genomic and phylogenetic evidence that the NAS genes uncovered in plant genomes are of microbial origin.

(6) Gene tree/species tree congruence could be yet another way to rule out contamination. It seems to be the case from looking at the supplementary figures, but it was not discussed in the manuscript.

Response: We agree, and had a statement reflecting this in the original text: "The phylogenetic relationships among mosses inferred from variation in their *NAS* genes (Supplementary Fig. 2, 3) reflect current concepts of diversification of these lineages [17], highlighting the occurrence of the *NAS* gene in the germ line and its vertical transmission, which further supports their effective integration in the genomes [18]." We further explicitly stated, respectively, in the legends to Supplemental Figures 2 and 3:

- *Gene tree of FunNAS gene inferred from their amino acid sequences, and reflecting the relationships among lineages of Funariidae, Dicranidae and "basal" Bryidae.*
- *Gene tree of the HypNAS and PolNAS gene inferred from their amino acid sequences, and reflecting the sister-group relationship of the clades of lineages harboring the HypNAS or the PolNAS, which suggests that the gene, which is of fungal origin (Supplementary Figure 1), was transferred from the ancestor of the Bryidae (minus Hedwigiales, Splachnales, and Bartramiales) to that of the sampled Polytrichaceae or vice versa.*

(7) Fig. 3 – That is not how Blasia should look like!

Response: Indeed, this was the only available icon publicly available for a liverwort. We have now created our own sketch and used that in Fig. 3. Note that we are also proposing to change the original version with distinguishing more the three main components (bacteria, fungi and land plants).

Reviewer #2 (Remarks to the Author):

The manuscript submitted by Dirick et al. provides some evidences of multiple cross-kingdom HGTs of nicotiamine synthase gene in land plants. I would like first to say that the results, as for the HGTs detection, are convincing, despite the fact that only one (phylogenetic incongruence, PI) among the three criteria commonly used for the detection of HGTs has been examined. Among the other two, the patchy distribution (PD) is somehow also met, although the authors do not really mention it, while the third one, the high similarity (HS) is not mentioned at all. The main reason for this is that the HGTs described here are very ancient and that among the three criteria, only the PI remains really useful.

Response: We assume that the reviewer refers to the three criteria by Leger et al. (BioEssays 2018, 40, e1700242) and referred to by Aubin et al. (cited in our manuscript): high similarity (HS), phylogenetic incongruence (PI) and patchy distribution in phylogenies (PD). While we do not report on sequence similarity per se, we do reflect patterns of similarity of plant NAS genes to various microbial NAS genes, via our phylogenetic reconstructions (Figs. 1 & S1). Our conclusion of independent transfers is explicitly based on the phylogenetic incongruence (gene versus species trees: Figs. 1, S1, S2 & S3) and patchy distribution in phylogenies (Figs. 1 & S1).

I also found the manuscript very well written and focused. I, however have two problems with it which, in my opinion, prevent its publication in NC as it stands:

1. - Orthology and gene replacement:
Throughout the manuscript, the authors use the term "orthologs", which is very confusing, unless I haven't fully understand their result: orthologs are homologs (with common ancestors) that remains at the same genomic location after an evolutionary split (eg speciation). Orthology is sometimes invoked when the function of the gene is conserved, although the homolog is located at a different locus. In such case, one should maybe better refer to functional homologs, but this is also misleading. Why this remark? Physcomitrella patens is known to integrate foreign DNA through homologous recombination (HR). Throughout the manuscript, when the authors invoke orthology, one wonders whether there are bona fide orthologous relationships among the horizontally transferred NAS, with conserved synteny, in particular whether the "replacement" that the authors mentioned in several instances has been obtained through HR or not, which is very important from an evolutionary perspective ?: if this is the case, frequent HGTs followed by HR could easily explain the propensity of bryophytes to diversify their gene space. If this is not, replacement could only be the result of a gain (through HGT) followed by a loss, but in this case, why isn't there a trace of the process, for instance in the form of pseudogenized NAS? The authors mention that they rule out the possibility of contamination by verifying that the genes flanking the NAS are bona fide plant genes. Are these the same in the different lineages?

Response:

- Regarding nomenclature: we understand the point made regarding the terminology used, but have failed to find an unambiguous term, and hence replace the word “ortholog” throughout the text simply with the word “gene”.
- Regarding homologous recombination: the replacement that we hypothesize of the FunNAS by a HypNAS, pertains to genes occurring at different genomic location (FunNAS integrated on Physcomitrium chr 8) is at a different locus than the HypNAS, which is the chromosome equivalent to Pp Chr18/21, and these chromosomes are not homeologs arising from an ancient whole genome duplication (see Lang et al., The Plant Journal, 2018, 93, 515–533). If HR is involved in the GT events, it might be through micro-homologies (for instance of repeated sequences/transposition sites), not by replacement of a NAS gene by a second one through HR at the same locus. In the example we describe (replacement of FunNAS by HypNAS), the most likely scenario is the integration of the HypNAS in the ancestor to the Bryidae (from Bryales to Hypnales in our phylogeny), bearing an FunNAS copy, followed by gene loss of the FunNAS copy.
- Regarding pseudogene: The loss of the FunNAS would likely date back to 300–360 million years, based on the chronograms[29] and hence be too ancient for a pseudogene to be retained.
- Regarding the flanking genes: As shown in Fig. S4, the flanking regions identified for various NAS genes differ among lineages of land plants bearing the gene.

2. - flowering plants.
If the authors ambition to publish in a high profile journal like NC, I think they should extend their analysis to the angiosperms and (to a lesser extent) the gymnosperms genomes available. I understand that some of the co-authors have an excellent expertise in Arabidopsis NAS, but the

lack of results on other angiosperms lineages is a big miss. Are there as many HGTs in flowering plants (for which HR is not possible) than in bryophytes?

Response: We have included a broad sampling of vascular plants, including ferns, gymnosperms, and many angiosperms in our initial NAS survey as shown in the phylogenies in Fig. S1 and S5. We have now enhanced the contrasts in the labelling of these Figures to enhance the visibility of the information. These figures illustrate our phylogenetic reconstruction, suggesting that a single HGT event is at the origin of NAS in Euphyllophytes, including in Angiosperms. (Figs. 2 and 3 HGT6, and Figs. S1 and S5).

Reviewer #3 (Remarks to the Author):

This is a very well written and conceived, highly original manuscript that considerably extend understanding of horizontal transfer of microbial genes in land plants by focusing on early diverging lineages (bryophytes). By broadening the search of the metallophore-synthesis NAS gene in mosses beyond the model species Physcomitrium patens, and including more than hundred new bryophyte genomes, the authors are able to show, for the first time, that the NAS genes have been acquired independently multiple times and from multiple microbial donors during the diversification of land plants. Thus, their findings challenge the current view of homology of microbial genes in plants, demonstrating instead that microbial gene exchange is much more dynamic than assumed. These are very important findings with the potential to provide a new layer of understanding to how plants conquered and diversified on land.
Although I am not an expert in all of the techniques, the methods appear sound, are clearly presented and easy to follow, and all the conclusions seem to be strongly backed by the results, which are also extremely well presented.
Overall, the manuscript was a pleasure to read and review. I found it intriguing that, within bryophytes, NAS genes are present only in mosses, which altogether lack mycorrhizal-like associations (I would be cautious of reports of these in Takakia), and only in one liverwort, Blasia pusilla, in the Blasiales, which stands out amongst liverworts not only for their symbioses with cyanobacteria but also as the only early divergent lineage that lack mycorrhizal-like associations. Perhaps the authors could also consider this latter point in their discussion.

Response: We have considered this for inclusion in the original manuscript but decided against for we were limited in space and also by the speculative nature of such discussion. The reluctance to develop this point reflects the incomplete correlation between mycorrhizae absence and NAS presence, as all lineages emerging from early splits in the moss tree (i.e., *Takakia*, *Sphagnum*, *Andreaeobryum* and *Andreaea*) lack both, and various liverworts lack mycorrhizae too, due to a secondary loss (Pressel et al., 2021, Bry. Div. Evo. 043 (1): 284–306).
We added the following in the text, to draw attention to the observed pattern, without, however, venturing into a speculative discussion.

Starting on line 218, we inserted the following:
"The NAS gene is currently only known, among bryophytes, from species lacking mycorrhizal associations, and may perhaps at least partially compensate for the absence of fungal symbionts by enhancing the capacity and efficiency of the internal distribution of metal ions."

Other than this, I have no suggestions as to make changes to the paper, which I find excellent. I was only able to spot a few minor typos that need correcting (e.g. Line 146: absences; Line 175:

addition; Line 247: analyses were implemented; Line 314: 21oC); although the reference list could benefit from some careful editing.

Response: Sorry for not catching these originally. All mistakes were corrected. Thank you. As for references, we used EndNote with Nature Communications ‘ settings to generate them. Nevertheless, we checked individual references and corrected the format where needed.

Answers to reviewer comments

Reviewer #1 (Remarks to the Author):

The authors have addressed most of my previous comments nicely. The statement “as in the case of the squalene synthase gene, transferred independently from cyanobacteria to bryophytes and to ferns.” Is not correct. It should be “squalene-hopene cyclase” and the donors of the bryophyte copies are not Cyanobacteria.

Response: corrected.

In the response to reviewers’ document, what is *Blasia pellucida*?

Response: corrected.

I still have concerns about data availability, but I will leave this to the editor to coordinate. When this paper is published, the genome data should be released as well.
Response: The data will be released with the original study.

Reviewer #2 (Remarks to the Author):

The authors have done a good job with addressing the points that I have raised, however they haven't revised the manuscript accordingly (see below).

"Previous response: We assume that the reviewer refers to the three criteria by Leger et al. (BioEssays 2018, 40, e1700242) and referred to by Aubin et al. (cited in our manuscript)" The three criteria were initially published by Wallau et al., GBE 2012.

Response: We agree that the criteria were first proposed by Wallau et al. However, as previously responded, we do not report on sequence similarity per se (see the original response next), hence we did not include a reference to Wallau et al.

"While we do not report on sequence similarity per se, we do reflect patterns of similarity of plant NAS genes to various microbial NAS genes, via our phylogenetic reconstructions (Figs. 1 & S1). Our conclusion of independent transfers is explicitly based on the phylogenetic incongruence (gene versus species trees: Figs. 1, S1, S2 & S3) and patchy distribution in phylogenies (Figs. 1 & S1)." This reply is OK to me but the text has not been appended accordingly.

Response: We did not modify the text, as the current paragraph structure and the figures (Figs. 1, S1, S2 & S3) reflect this rationale.

"Regarding homologous recombination: the replacement that we hypothesize of the *FunNAS* by a *HypNAS*, pertains to genes occurring at different genomic location (*FunNAS* integrated on Physcomitrium chr 8) is at a different locus than the *HypNAS*, which is the chromosome equivalent to Pp Chr18/21, and these chromosomes are not homeologs arising from an ancient whole genome duplication (see Lang et al., The Plant Journal, 2018, 93, 515–533). If HR is involved in the GT events, it might be through micro-homologies (for instance of repeated sequences/transposition sites), not by replacement of a NAS gene by a second one through HR at the same locus. In the example we describe (replacement of *FunNAS* by *HypNAS*), the most likely scenario is the integration of the *HypNAS* in the ancestor to the Bryidae (from Bryales to Hypnales in our phylogeny), bearing an *FunNAS* copy, followed by gene loss of the *FunNAS* copy." Why is this not mentioned in the new version?

Response: The mechanism leading to the loss of the *FunNAS* by a *HypNAS* is not known, and a discussion of such seems too speculative to be included in the manuscript.

"Regarding pseudogene: The loss of the *FunNAS* would likely date back to 300–360 million years, based on the chronograms29 and hence be too ancient for a pseudogene to be retained." I am ok with this. But why is this not mentioned in the new version?

Response: We added the sentence "Evidence of pseudogenization of the *FunNAS* in any Bryidae genome is lacking, most likely due to the loss dating back to about 200 million years ago [15].
